# Lack of optimistic bias during social evaluation learning reflects reduced positive self-beliefs in depression and social anxiety, but via distinct mechanisms

**DOI:** 10.1038/s41598-024-72749-6

**Published:** 2024-09-28

**Authors:** Janina A. Hoffmann, Catherine Hobbs, Michael Moutoussis, Katherine S. Button

**Affiliations:** 1 Department of Psychology, University of Bath, Bath, BA2 7AY UK; 2 Department of Imaging Neuroscience, Institute of Neurology, University College London, London, UK

**Keywords:** Learning and memory, Reward, Social behaviour, Anxiety, Depression, Human behaviour

## Abstract

Processing social feedback optimistically may maintain positive self-beliefs and stable social relationships. Conversely, a lack of this optimistic bias in depression and social anxiety may perpetuate negative self-beliefs and maintain symptoms. Research investigating this mechanism is scarce, however, and the mechanisms by which depressed and socially anxious individuals respond to social evaluation may also differ. Using a range of computational approaches in two large datasets (mega-analysis of previous studies, *n* = 450; pre-registered replication study, *n* = 807), we investigated how depression (PHQ-9) and social anxiety (BFNE) symptoms related to social evaluation learning in a computerized task. Optimistic bias (better learning of positive relative to negative evaluations) was found to be negatively associated with depression and social anxiety. Structural equation models suggested this reflected a heightened sensitivity to negative social feedback in social anxiety, whereas in depression it co-existed with a blunted response to positive social feedback. Computational belief-based learning models further suggested that reduced optimism was driven by less positive trait-like self-beliefs in both depression and social anxiety, with some evidence for a general blunting in belief updating in depression. Recognizing such transdiagnostic similarities and differences in social evaluation learning across disorders may inform approaches to personalizing treatment.

**Supplementary Information:**

The online version contains supplementary material available at 10.1038/s41598-024-72749-6.

## Introduction

Depression and social anxiety are highly co-morbid disorders that significantly contribute to the global burden of disease^1,2^. Both are characterized by negative views of the self^3,4^ and widespread social difficulties^5,6^. A better understanding of the social cognitive biases sustaining negative self-beliefs in depression and social anxiety across the population can inform our knowledge of the mechanisms of these disorders, and is likely to advance psychosocial, and possibly neurobiological, interventions^7^.

Social interactions are dynamic and complex, with social behaviors (e.g., a smile, praise) and social inferences (e.g., “this person likes me”) dependent on both feedback from others as well as its subjective interpretation. Perceptions of the self are largely informed by how we believe others view us^8^. During social interactions, healthy individuals update their self-image more strongly after positive versus negative feedback^9^. These optimistic social biases are believed to benefit mental health by increasing confidence and self-esteem^10^. In contrast, individuals with depression and social anxiety lack optimistic social biases. They tend to believe that others judge them negatively^11,12^ and underestimate their social skills^13,14^. A lack of optimism when processing social feedback likely maintains maladaptive negative self-beliefs.

Maladaptive negative views of the self are transdiagnostic across depression and social anxiety but may be maintained via different learning mechanisms. According to cognitive-behavioral theories, negative beliefs coalesce in negative self-schema which when activated, act as a prism through which incoming sensory information is processed^3^. Individuals with social anxiety fear negative evaluation, and hold schema centering on maladaptive beliefs of their social skills (e.g., “I am stupid”) and the expectations of others (e.g., “They will criticize me”) which lead to the generation of negative mental images of how they appear to others^4^. These negative self-beliefs enhance responsivity to socially threatening information which then reinforces negative expectations in a cyclical process^4,15^. Supporting this idea, individuals experiencing social anxiety show greater sensitivity to negative facial emotions^16,17^ and are less often biased towards positive social information compared to healthy volunteers^18–20^. Following schema theories of social anxiety, we would therefore expect socially anxious individuals to be particularly sensitive to learning from negative social evaluations about the self.

A similar causal role for negative self-beliefs is proposed by cognitive theories of depression but here the schema is less focused on social situations and relates more to general feelings of low self-worth and lack of optimism for the world and the future^3^. Depression is also characterized by anhedonia, a general loss in the ability to feel pleasure, potentially manifesting in reduced sensitivity to reward^21,22^. Individuals with depression report social anhedonia in the context of social interactions – a loss of interest or pleasure in social interactions^23^, which may divert attention and learning from social evaluations. Supporting the idea that depressed individuals generally learn slower from positive feedback^24^, individuals experiencing depression are less prone to optimistic updating biases. Depressed individuals do not update their beliefs more following good compared to bad news^25^, are less likely to integrate novel positive information into expectations about their own performance^26^, and revise negative interpretations of social situations less flexibly after receiving disconfirmatory positive feedback^27,28^. Further, when anticipating reward, depressed individuals are less willing to expend cognitive or physical effort for reward^29^ and show a reduced activation in the striatum, an area of the brain critical to reward systems^30^. Although some research has reported evidence that depressed individuals are more sensitive to punishment^31^, impairments in reward-based processing are more reliably observed^21,22^. However, some studies also failed to replicate the finding that individuals with depression differ from healthy controls in their sensitivity to rewards, suggesting that the anhedonia hypothesis is not unequivocally supported^32,33^. We suggest that this reduced sensitivity to reward may result in blunted reactivity to positive social feedback in the context of social evaluation learning which may perpetuate negative self-schema through reduced exposure to more positive views of the self.

Cognitive theories of depression and social anxiety conceptualize the formation and maintenance of biased beliefs as self-reinforcing loops, in which initial negative mental images of the self (biased beliefs) are upheld through a less optimistic interpretation of social interactions (biased updating). However, mechanistic evidence is scarce^7^. It is thus important to distinguish initial beliefs from persistent maladaptive learning patterns. This has been difficult to achieve using standard descriptive measures of social evaluation learning, such as error rates, which confound these distinct socio-cognitive mechanisms. Computational learning models have been widely used to distinguish how biased initial beliefs, variations in learning speed, and preferential reward processing alter the learning process^22,24,34^. Associative learning models, the standard modeling framework in computational psychiatry^35,36^, propose that individuals adjust the perceived value of themselves and others during interactions depending upon the prediction error, that is the discrepancy between their expectations and the social feedback. Belief updating models, in contrast, represent beliefs about the self and others as probabilities and update these probabilities through a process of evidence accumulation. While initial computational work identified a higher learning rate for negative information as the driving mechanism behind faster learning from negative feedback in socially anxious persons^37,38^, our recent computational work pointed towards a more influential role of reduced positive self-beliefs^34^. This raises the question if social anxious persons initially activate negatively biased trait beliefs, leading to a biased interpretation of how others view them, or if also each feedback from others is interpreted more negatively, as biased updating processes suggests. Further, it is unknown if the same belief and updating biases explain deficits in social evaluation transdiagnostically, that is in social anxiety and depression. A general issue across research fields is the reliability of findings given the focus on novel research questions and the reliance on small sample sizes^39^. Systematic comparisons between associative and belief-based modeling approaches are scarce. Here, we aimed to address this by investigating the computational mechanisms underpinning social evaluation learning in large datasets and to investigate to what degree the same mechanisms generalize from social anxiety to low mood.

We aimed to examine learning of social evaluations in individuals experiencing varying levels of social anxiety and depression, to establish the reliability of previously reported effect estimates and to investigate transdiagnostic and specific effects. Using two independent datasets to establish the reproducibility and generalizability of our findings (see Methods and Materials), we tested two pre-registered hypotheses [ https://osf.io/r3peu ]. “*Both depression and anxiety will be associated with more negative social evaluation learning styles relative to those with lower symptom scores*” (H1.1). Specifically, depression and social anxiety would be associated with a reduced optimistic bias in learning social evaluations. In terms of disorder-specific patterns, social anxiety would be associated with “*fewer errors before learning the ‘I am disliked’ rule*” but depression would be “*associated with reduced sensitivity to social reward*”, as indicated by “*a greater number of errors made before learning the ‘I am liked’ rule*” (H1.2). To elucidate the mechanisms underpinning how social anxiety and depressed mood alters feedback processing and learning, we also tested pre-registered hypotheses about computational processes. We *“hypothesized that these differences in learning will be reflected in computational parameters and especially ‘learning rates’”* (H2.1). Specifically, we hypothesized that “*the socially anxious would be less certain of their own positive traits*,* and consequently more sensitive to being swayed by social threat*,* whereas the more depressed one is*,* the more they are certain about their own negative traits and hence more resistant to learning that they are liked”* (H2.2). We employed belief updating models to test these hypotheses and tested whether associative learning models better accounted for our data.

## Results

**Fig. 1:**
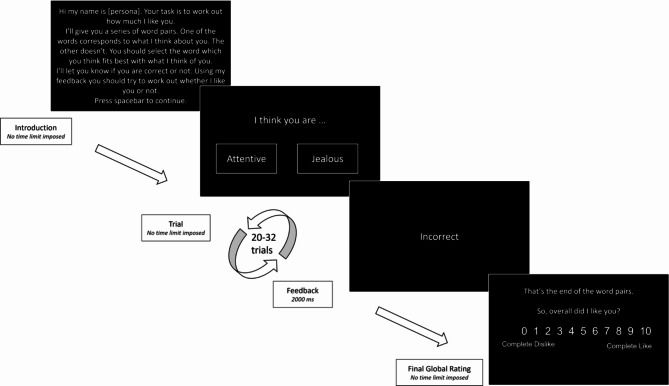
Example of the self-referential condition in the social evaluation learning task. Participants selected in each trial a positive or a negative evaluative word to predict whether the computer liked them (self-referential condition) or a fictional other (other-referential condition). The proportion of trials with ‘correct’ feedback to a positive word reflected three possible rules: 60–80% positive feedback for positive rules, 20–40% positive feedback for negative rules, and 50% positive feedback for ‘neutral’ rules (80-20-50 for the pre-registered study). At the end of each referential condition-rule block participants provided a global rating of how much the computer liked the person being learnt about (complete dislike 0–10 complete like).

We conducted a mega-analysis on data from nine studies previously collected in our research group (‘Mega-analysis’ Dataset) to establish a robust link between the learning of social evaluations and psychopathological traits. In all studies, learning of social evaluations was measured using a two-alternative forced choice probabilistic learning task (Fig. 1, task characteristics are outlined in SI Study characteristics). We measured depression severity with the Patient Health Questionnaire (PHQ-9)^40^ and social anxiety with the Brief Fear of Negative Evaluation Scale (BFNE)^41^. Next, we assessed the reliability of our findings in a new dataset (‘Preregistered’ Dataset). Finally, we developed computational models of social evaluation learning on the dataset Mega-analysis (see Methods): we equipped an associative learning model and a belief-updating model with the most parsimonious set of features necessary to account for biased processing of social feedback in depression and social anxiety. We then identified the best belief-based and associative models in terms of accounting for behavior, irrespective of psychopathology. Finally, we tested the relationship between model parameters and psychopathological measures.

**Table 1:** Sample characteristics and errors in the social evaluation learning task by dataset. Greater bias scores indicate better learning of the positive rule relative to the negative rule.

	Mega-analysis	Preregistered		
Sample characteristics				
Total N^a^	450	807		
Age, M (SD)	23.6 (7.2)	35.1 (12.6)		
Gender, N (%)				
Female	303 (75.4)	455 (56.7)		
Male	98 (24.4)	348 (43.3)		
Ethnicity, N (%)^a^				
White	235 (74.1)	698 (86.5)		
Ethnic minority	82 (25.9)	109 (13.5)		
Occupation, N (%)^a^				
Employed	80 (22.9)	524 (64.9)		
Student	260 (74.5)	134 (16.6)		
Other	9 (2.6)	149 (18.5)		
PHQ-9, M (SD)	6.2 (5.2)	6.8 (5.3)		
BFNE, M (SD)	37.2 (11.1)	37.9 (11.0)		
*Social evaluation learning*				
Errors to criterion, M (SD)	**Self**	**Other**	**Self**	**Other**
Positive	4.9 (4.6)	5.1 (4.7)	4.0 (5.1)	4.2 (5.4)
Negative	7.5 (6.0)	7.1 (5.3)	8.7 (8.5)	7.5 (7.3)
Bias Scores, M (SD)	2.6 (7.6)	2.0 (6.6)	4.7 (10.2)	3.3 (9.1)

PHQ-9 = Patient Health Questionnaire; BFNE = Brief Fear of Negative Evaluation.

Errors to criterion = average number of errors made before eight-consecutive rule-congruent responses.

^a^ Not all studies in the Mega-analysis assessed the same demographic information (SI Study characteristics). Percentages are calculated across non-missing entries.

### Social evaluation learning predicts depression and social anxiety

Across datasets, participants demonstrated an ‘optimistic bias’, making fewer errors on the positive, ‘like’ rule relative to the negative, ‘Dislike’ rule (*p* < .001 in both datasets, Table 1 for summary statistics, detailed results and figures are reported in SI Learning errors in social evaluation). Mixed-effects linear regression models indicated that this tendency was more pronounced when learning about the self in the Preregistered study (referent x rule: *p* < .001), but not in the Mega-analysis (*p* = .331).

**Fig. 2:**
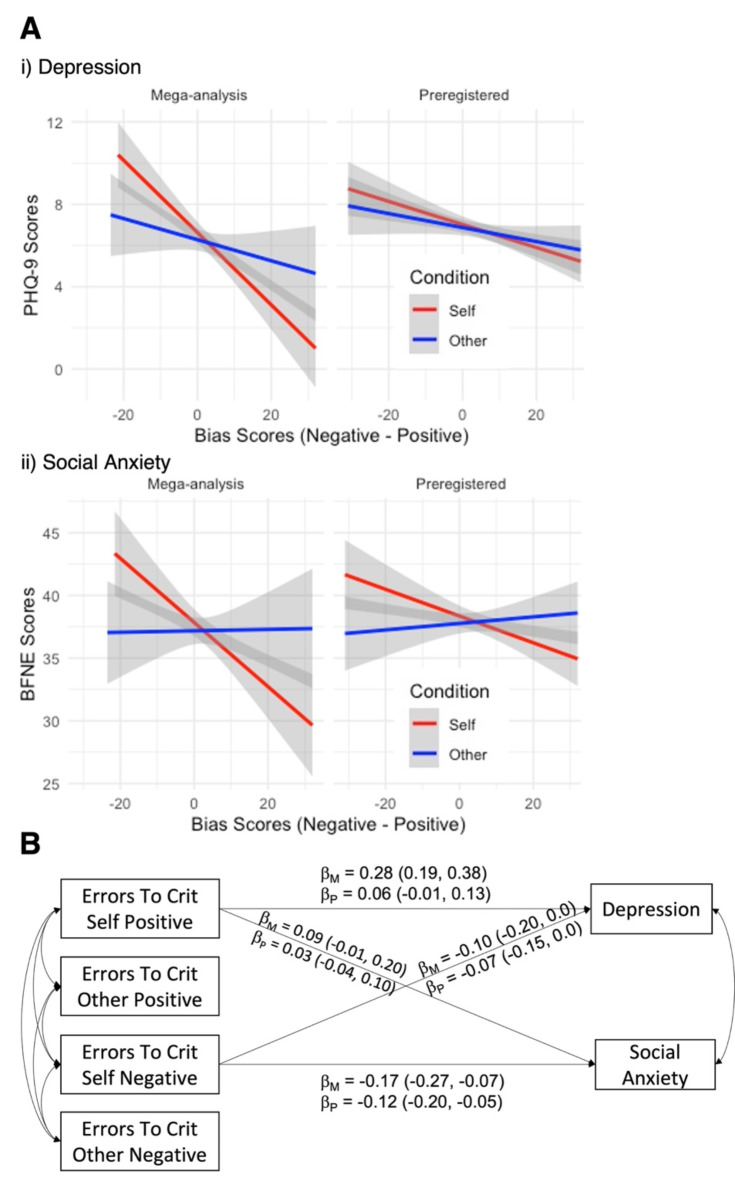
Depressive symptoms and social anxiety correlate with optimistic bias and errors to criterion in social evaluation learning. (A) Relationship between optimistic bias (fewer errors on the positive rule) in the social evaluation learning task, depression (PHQ-9) and social anxiety (BFNE). Optimistic bias about the self decreased as PHQ-9 and BFNE scores increased. (B) Structural equation modelling of depressive symptoms and social anxiety statistically predicted by errors to criterion in each referential-rule combination. Social anxious participants committed fewer errors to criterion for the negative rules about the self in the Mega-analysis (β_M_) and the Preregistered (β_P_) dataset; depressive symptoms were not unambiguously associated with the number of errors to criterion.

Structural equation modelling provided strong evidence that depressive symptoms and social anxiety decreased as optimistic bias increased, supporting H1.1 (Fig. 2A). In the Mega-analysis, participants who made fewer errors on the positive rule than the negative rule reported fewer depressive symptoms (β = -0.25, 95% CI = [-0.34, -0.15], *p* < .001) and reduced social anxiety (β = -0.19, 95% CI = [-0.28, -0.10], *p* < .001). These effects were specific to processing information about the self; there was no significant relationship between processing information about others and symptomatology (SI Social evaluation and psychopathological traits). These results were qualitatively replicated in the Preregistered dataset, albeit attenuated (depression: β = -0.10, 95% CI = [-0.18, -0.03], *p* = .007; social anxiety: β = -0.13, 95% CI = [-0.20, -0.05], *p* < .001; between-study comparison: Δχ^2^(4) = 10.9, *p* = .03, RMSEA = 0.05).

We expected that participants with higher social anxiety would have fewer errors to criterion for the negative rule about the self (i.e., social threat), whereas persons with more depressive symptoms would have greater errors to criterion for the positive rule about the self (i.e., social reward, H1.2). We tested these hypotheses in a structural equation model that predicted depressive symptoms and social anxiety with errors to criterion in each of the referential condition-rule combinations (Fig. 2B). Supporting our hypothesis H1.2 on social anxiety, greater social anxiety was associated with reduced errors to criterion for the negative rule about the self in both datasets (Mega-analysis: β = -0.17, 95% CI = [-0.27, -0.07], *p* = .001; Preregistered: β = -0.12, 95% CI = [-0.20; -0.05], *p* = .001), but unrelated to errors to criterion for the positive rule about the self (Mega-analysis: β = 0.09, 95% CI = [-0.01, 0.20], *p* = .067; Preregistered: β = 0.03, 95% CI = [-0.04; 0.10], *p* = .414). Our hypothesis related to depressive symptoms was only partially supported (H1.2). In the Mega-analysis, we found strong evidence that more depressive symptoms were associated with increased errors to criterion for the positive rule (β = 0.28, 95% CI = [0.19, 0.38], *p* < .001). Like social anxiety, depression was also associated with reduced errors to criterion for the negative rule (β = -0.10, 95% CI = [-0.20; -0.00], *p* = .048). However, in the Preregistered study, we observed effects in the same direction but overlapping with the null to a small extent (Self-Positive: β = 0.06, 95% CI = [-0.01; 0.13], *p* = .093; Self-Negative: β = -0.07, 95% CI = [-0.15; 0.00], *p* = .056). In line with this finding, a comparison of effect sizes between studies suggested that all effects were less pronounced in the Preregistered dataset (between-study comparison, Δχ^2^(8) = 21.0, *p* = .007, RMSEA = 0.05).

### Cognitive modelling stresses the role of belief updating and trait beliefs in social evaluation learning

A large initial search for plausible mechanisms underpinning social evaluation learning revealed six promising candidate models in associative learning and belief updating (SI: Computational modelling of social evaluative learning). In a model evaluation step, we identified the best associative learning model, the best belief updating model, and the globally best reinforcement learning model among these candidates. Model comparisons following our original protocol clearly demonstrated that belief updating models described social evaluative learning on average better than associative learning models and this advantage replicated across datasets and fit measures. The best belief updating model encompassed a tendency to choose the positive option more often than the negative option, expressed as a stable trait belief. Trait beliefs were stronger in the self-referential compared to the other- referential conditions. Further, the best belief updating model included separate parameters to capture the speed of updating and a decay of evidence in memory. Crucially, the distinction between trait beliefs for the self and others allows us to test the hypothesis that socially anxious (or depressed) persons hold stable biased trait beliefs about the self, but not about other persons. However, the best belief updating model does not differentiate speed of updating for learning about the self from learning about others. Therefore, we had to reformulate Hypothesis H2.2 and instead explored whether depressed (or socially anxious) individuals revise their interpretations of any social evidence less flexibly^27,28^ and thus *”depression (or social anxiety) biased the accumulation of social evidence about self and others”* (H2.2). In the remainder we focus our analysis on this best belief updating model (named Decay Trait Belief SO); associative learning models largely supported the same conclusions and results are reported in SI Associative learning in social evaluation and psychopathological traits.

**Fig. 3:**
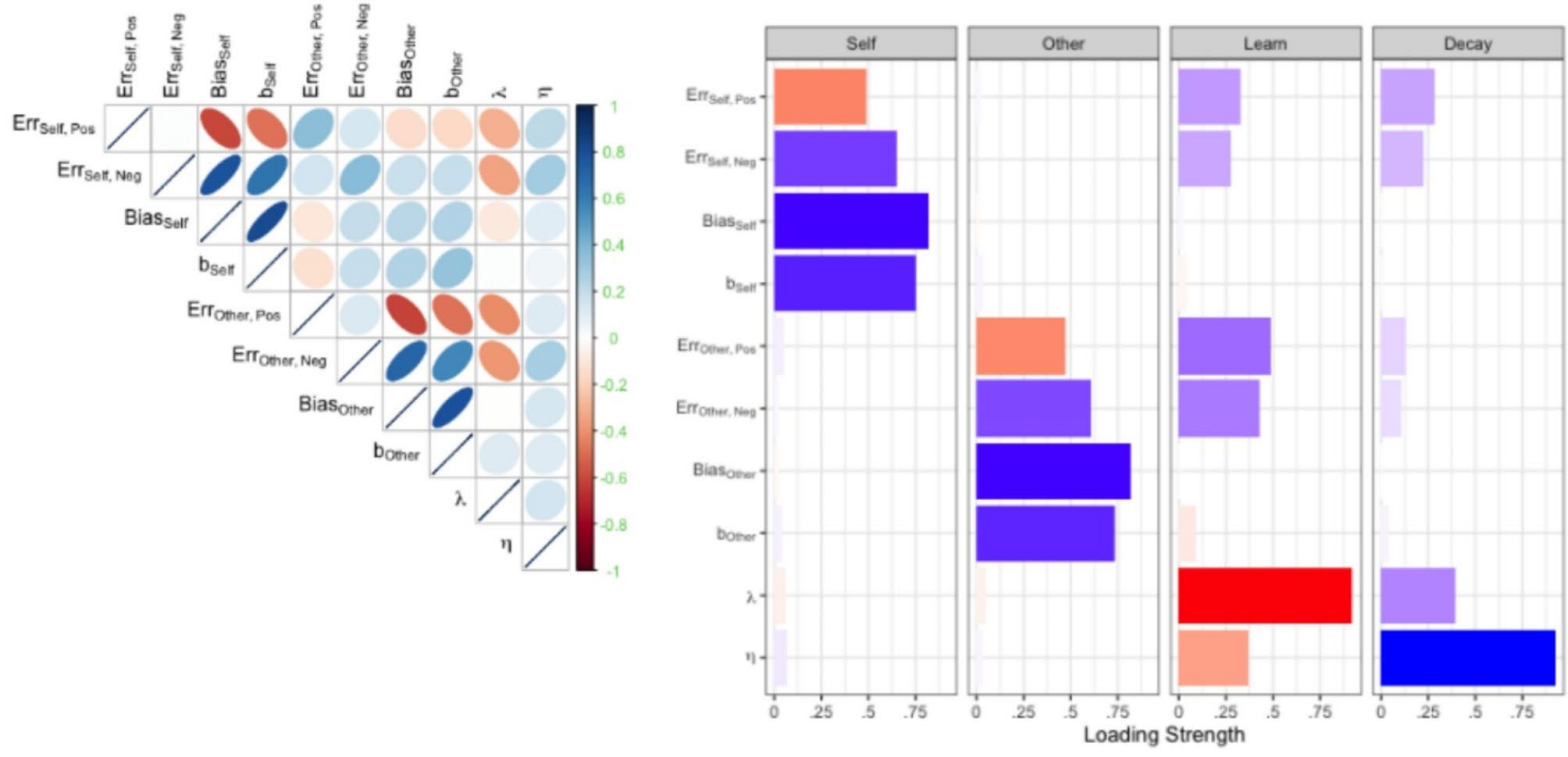
Relationship between computational modelling parameters, optimistic biases, and learning errors. Left panel: Correlations between model parameters ($\:{\lambda\:,\:\eta\:,\:b}_{\text{Self}}{,\:b}_{\text{Other}}$) of the belief updating model, errors to criterion in each referential-rule condition (Err), and optimistic bias scores (BiasSelf, BiasOther). Right panel: Factor loadings in a principal component analysis for the best belief updating model. Blue colors indicate positive correlations (loadings); red colors indicate negative correlations (loadings).

We assessed the construct validity of belief updating parameters with a principal component factor analysis using errors to criterion and optimistic bias as additional independent variables (Fig. 3, SI Belief updating in social evaluation and psychopathological traits). The variability in model parameters and scores was sufficiently explained by a 4-factor solution in belief updating (86.8% of the variance explained) according to eigenvalues and a parallel analysis. In belief updating, trait-like beliefs about the self $\:{\:b}_{\text{Self}}$reflected the optimistic bias in the self-referential condition, whereas trait beliefs about others $\:{b}_{\text{Other}}$ primarily reflected optimistic bias in the other condition. Stronger updating rates for beliefs $\:\lambda\:$ related negatively to learning errors, as might be expected but the forgetting of evidence $\:\eta\:$ had weak descriptive counterparts. Taken together, model parameters tuned four types of cognitive mechanism: processing of positive self-beliefs, processing of positive other-beliefs, and the impact of evidence, globally and specifically connected to the self.

### Positive self-beliefs are consistently associated with depression and social anxiety, less so learning rates or styles

**Fig. 4:**
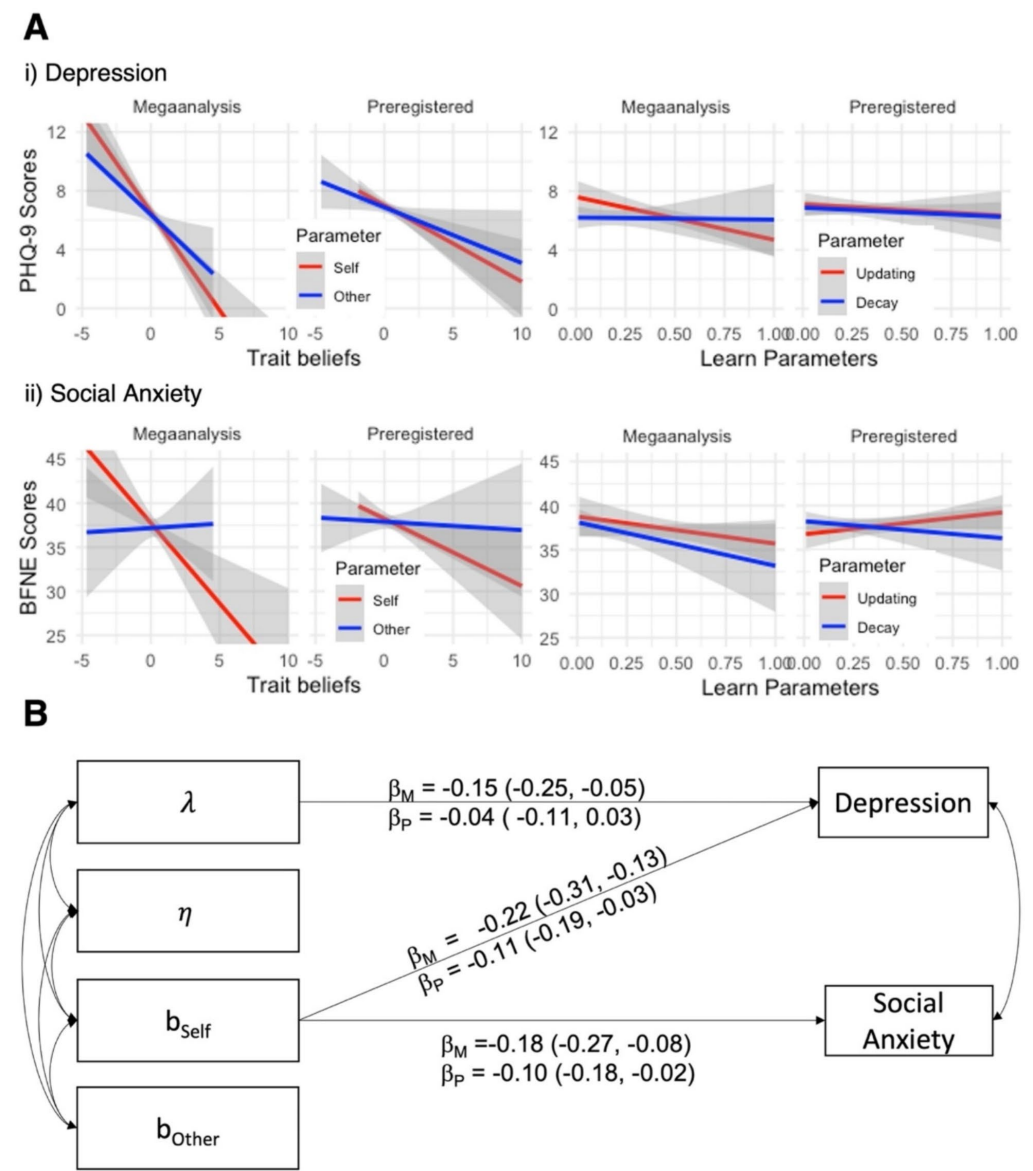
Depressive symptoms and social anxiety correlate with trait beliefs, but less with learning rates in social evaluation learning. (A) Relationship between belief updating parameters estimated in the social evaluation learning task and depressive symptoms (PHQ-9, i) and social anxiety (BFNE, ii). Higher trait beliefs for the self *b*_Self_ (or the other *b*_Other_) indicate that participants believed others perceived them (or another person) positively. Higher updating rates *λ* (or decay rates *η*) indicate that participants faster updated their beliefs (or faster erased previously held beliefs). Higher trait beliefs about the self were consistently associated with fewer depressive and social anxiety symptoms. (B) Structural equation modelling of depressive symptoms and social anxiety as predicted by computational parameters estimated within the belief updating model. Social anxious participants and depressed participants were more likely to believe that others perceived them positively *b*_Self_, faster updating *λ* was not unambiguously associated with depressive symptoms.

To test our hypotheses about the role of self-beliefs, other beliefs, and the impact of evidence in socially anxious or depressed participants, we correlated each model parameter with social anxiety and depression scores. Figure 4A illustrates how the scores were consistently negatively correlated with positive trait beliefs about the self, indicating that individuals with higher social anxiety and depressive symptoms were less often inclined to believe that others perceived them positively. In addition, higher PHQ-9 scores were associated with lower trait beliefs about fictitious others, indicating that individuals with higher levels of depressive symptoms also less often believed that others are evaluated positively. Finally, individuals with more depressive symptoms updated their beliefs somewhat more slowly, as indicated by lower updating rates, but this relationship appeared to be attenuated in the Preregistered dataset.

Structural equation modelling in both datasets supported the hypothesis *“that these differences in learning will be reflected in computational parameters and especially ‘learning rates’”* (H2.1, Fig. 4B). Positive trait beliefs about the self *b*_Self_ were predictive of lower levels of social anxiety (Mega-analysis: β = -0.18, 95% CI = [-0.27, -0.08], *p* < .001; Preregistered: β = -0.10, 95% CI = [-0.18; -0.02], *p* = .013) and better mood (Mega-analysis: β = -0.22, 95% CI = [-0.31, -0.13], *p* = .001; Preregistered: β = -0.11, 95% CI = [-0.19; -0.03], *p* = .005). We found partial evidence for the idea that learning rates differ between depression and social anxiety, however this seemed to reflect a general blunting in updating beliefs following social evidence in depression, rather than being specific to positive or negative information (H2.2). In the Mega-analysis, slower updating of beliefs λ corresponded to more depressive symptoms (β = -0.15, 95% CI = [-0.25, -0.05], *p* = .002). This relationship did not replicate in the Preregistered dataset (β = -0.04, 95% CI = [-0.10, 0.03], *p* = .251), although relationships between model parameters and symptomatology did not differ strongly across studies otherwise (between-study comparison, Δχ^2^(8) = 14.8, *p* = .061, RMSEA = 0.04).

**Fig. 5:**
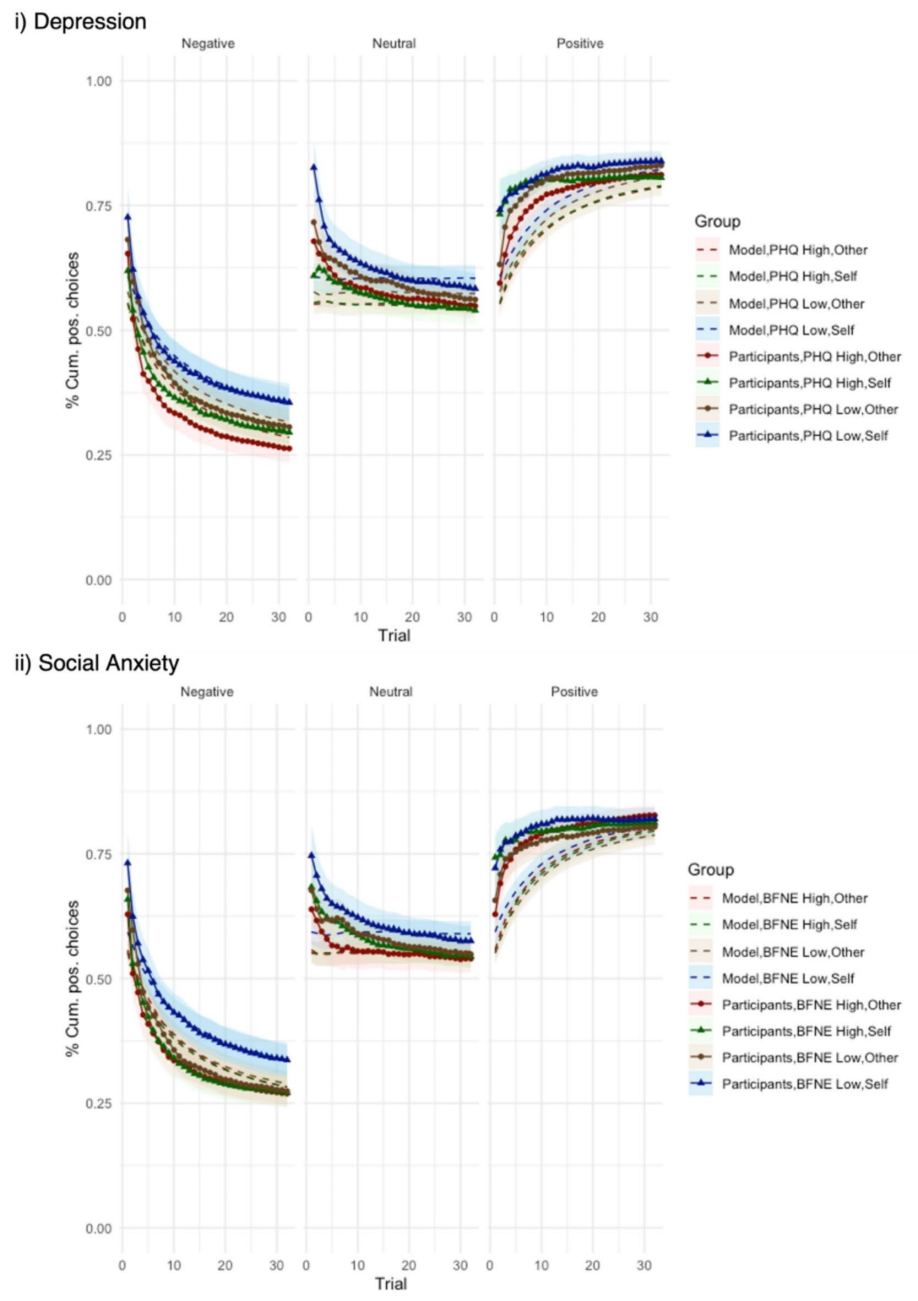
Social evaluation learning of participants and predicted by the belief updating model. Cumulative proportion of positive evaluations chosen by participants (solid lines with markers) against predictions of the belief updating model (dashed lines) for the 25% of individuals with the (i) highest and lowest depressive symptoms (PHQ scores) and (ii) highest and lowest social anxiety (BFNE scores). Individuals with high depressive symptoms (or high social anxiety) learn the negative rule faster than individuals with low depressive symptoms (or high social anxiety), but learn the positive rule more slowly. The belief updating model reproduces these behavioral trends, although it picks up the rules, particularly the positive rule at a slower pace.

To illustrate differences in social evaluation learning for individuals with low or high levels of depression (or social anxiety), we plotted separately the learning pathways for the 25% of individuals with the lowest and the highest PHQ scores (or BFNE scores, respectively) as well as their model predicted learning pathways (Fig. 5). Model predictions and participants’ actual learning curves clearly demonstrate that participants with high levels of depression (or social anxiety) learn the positive rule more slowly compared to participants with low levels of depression, but pick up the negative rule faster, indicating more pessimistic trait beliefs about the self across all rules.

In a final step, we assessed the degree to which one learning style, associative learning or belief updating, explains differences in psychopathological traits. To this goal, we computed for each participant the evidence for the best associative learning model compared to the best belief updating model as a BIC weight^20^ and correlated this BIC weight with BFNE and PHQ-9 scores, respectively. Associative learning was not consistently associated with social anxiety (Mega-analysis: *r* = − .03, 95% CI = [-0.06, 0.13], *p* = .001; Preregistered: *r* = − .07, 95% CI = [-0.14; − 0.00], *p* = .039), nor with depressive symptoms (Mega-analysis: *r* = .08, 95% CI = [-0.02, 0.18], *p* = .110; Preregistered: *r* = − .01, 95% CI = [-0.08; 0.06], *p* = .741). Taken together, these results replicate our previous analyses^34^ and suggest that belief updating models better explain the relationship between social evaluation learning and depression and social anxiety.

## Discussion

Individuals experiencing depression and social anxiety are believed to react more sensitively to negative social feedback and less sensitively to positive social feedback, reinforcing negative views of the self^4,15,42,43^. It remains unclear, however, to what extent social feedback is processed differentially in depression and social anxiety, and which cognitive mechanisms are most important in social evaluation learning within different dimensions of symptomatology. We therefore applied a transdiagnostic approach^23^ to investigate learning of positive and negative social evaluations in individuals experiencing varying levels of depression and social anxiety.

Our findings support previous work in suggesting that optimistic trait-like beliefs about the self are associated with better mental health^9,10^. On average, participants displayed optimistic biases in social evaluation, making fewer errors on positive rules relative to negative rules. The greater the symptoms of depression and social anxiety participants reported, the more errors they committed when learning the positive relative to the negative rule. Computational modelling indicated that optimistic trait beliefs best explained the relative advantage of learning the positive compared to the negative rule, consistent with the idea that individuals activate positive schemata in social interactions^34^. Further, computational modeling supported the idea that people hold distinct schemata or trait beliefs about the self and other persons. Reduced optimistic trait beliefs about the self independently predicted social anxiety levels and depressive symptoms, in a transdiagnostic manner. These effects were specific to optimistic beliefs about the self; we did not find evidence that beliefs about others were associated with either social anxiety or depression. This suggests that depression and social anxiety are characterized by trait-like beliefs of weaker positive evidence about the self, in the context of social evaluations. Such relatively negative self-schemas may be important in maintaining the negative self-image characteristic of both disorders^18,44,45^.

We found some evidence that maladaptive beliefs in depression and social anxiety are upheld via distinct learning mechanisms. Social anxiety was robustly associated with better learning of the negative rule about the self in both datasets, but not with changed learning of the self ‘like’ rule. Matching these descriptive results, computational belief updating models suggested that participants with higher levels of social anxiety possess less positive trait beliefs about the self. Surprisingly, we don’t find evidence for biases in evidence accumulation (updating parameter *λ*), forgetting of evidence (memory decay $\:\eta\:$) or in learning rates in social anxiety (H2.1). This finding dovetails computational work that has identified biased self-beliefs as the key mechanism underpinning faster learning from negative feedback in social anxiety^34^, pointing towards a limited role of biased updating processes. Participants with more depressive symptoms, in contrast, made fewer errors when learning the negative ‘dislike’ rule about the self and more errors when learning the positive ‘like’ rule about the self. Matching these descriptive results, belief updating models suggested that depression was also characterized by less positive trait beliefs about the self and – less reliably – slower updating of beliefs in general. The latter finding supports previous work stressing the notion that depressed individuals may revise negative interpretations of social situations less flexibly^27,28^. Jointly considered, these findings hint at a differential role of social approval in social anxiety and depression. In line with cognitive theories, social anxiety is partly driven by an increased sensitivity to socially threatening information which reinforce negative beliefs about the self^4^. We did not find evidence that socially anxious participants made more errors learning the ‘like’ rule about the self as indicative of a fear of positive evaluations^46^, nor that social anxious participants updated their self-concept faster after selecting a positive or negative word (SI: Associative learning of social evaluation and psychopathological traits). Depression, in contrast, may be characterized by blunted accumulation of social evidence (*λ* in Fig. 4). This partially supported finding is consistent with the present but weak effects of reduced reward sensitivity in depression and may speak to current clinical approaches that re-cast depression as reduced (mindful) awareness of social information^47^. In the real world, lacking full awareness of social feedback may increase the probability of reciprocal negative behaviors from others, and contribute to relationship difficulties and a lack of social support commonly associated with depression^16^.

## Clinical implications

Our findings give hope that learning within social interactions may be a potentially beneficial target for improving treatments for depression and social anxiety if they generalize to help-seeking patients. Our results also suggest that tailoring treatments to target beliefs about the self as well as specific learning difficulties of individual patients may be more effective than a general approach. Treatments for more social anxious patients may profitably focus on increasing positive trait beliefs about the self by reducing attention to negative self-beliefs in the context of social situations, thereby reducing sensitivity to negative social feedback. Treatments for more depressed patients may also seek to restore the dampened updating rate by increasing mindful attention to the affective quality of social feedback, thereby increasing the cumulative impact of socially rewarding information. In line with this idea, recent therapeutic studies provide initial evidence that informing participants with major depressive disorder about the value of positive feedback may aid to boost expectations about the self^48^. Our finding that positive learning biases towards a fictional ‘other’ were maintained in individuals with high social anxiety and depression support a technique commonly used in cognitive therapies; asking patients to challenge their maladaptive beliefs by considering a scenario from a third-person perspective^49^. Our findings suggest that cognitive therapies for depression and social anxiety should continue to use this technique to address unhelpful cognitive biases. Finally, psychoeducation is a crucial component in most psychological therapies and brief, passive psycho-educative interventions offer a low-barrier initial step towards improving mental health^50^. Therefore, educating low mood individuals about activated negative self-schemata and reduced mindful awareness of social feedback might serve as a brief, inexpensive initial intervention.

## Strengths and limitations

We took a rigorous methodological approach and assessed the reliability of our findings first in a large re-analysis of nine previous studies and next established their replicability in an independent online study. Relatively large samples in both datasets ensured adequate statistical power to detect small effects. Our computational modelling helped replicate and refine previous work on the cognitive mechanisms behind learning by accumulation of salient, social evidence, rather than staying at a descriptive level. Future research may seek to replicate our findings in independent studies and generalize these insights across different research paradigms studying social evaluation learning and different measures of social anxiety and depressive symptoms.

Our study design was not suited to determine causal relationships. Whilst we found strong evidence of a cross-sectional relationship between social evaluation learning, depression, and social anxiety, we limited the assessment of social anxiety to fear of negative evaluation. Social anxiety is, however, characterized by a high general fear of evaluation, often measured as fear of positive and negative evaluation^51^, suggesting an interesting route for future work. Further, it is possible that social cognitive processes are symptomatic as well as perpetuating of these disorders (feedback loops). Future longitudinal and interventional research is required to dissect this possibly circular causality.

Psychopathological traits showed a weaker association to social evaluation learning in the Preregistered dataset compared to our Mega-analysis, as indicated by between-study comparisons. Some studies within the Mega-analysis used extreme group designs and selectively recruited individuals with very high or low levels of symptoms which may contribute to larger effect estimates. Further, we changed the methodology from lab-based assessment in the Mega-analysis to online assessment in the Preregistered study. Although cognitive testing across lab and online settings has been claimed to produce comparable results^52^, remote participants may report more often depressive symptoms^53^ or get more easily distracted. Demographic differences and recruitment policies may also play a role (Table 1; Table A1). Further research optimizing cognitive online tasks for depressed and socially anxious individuals would be helpful in contextualizing our results, and support future on-line treatments based on social interactions.

## Conclusions

Across two independent datasets we found that participants with greater social anxiety and depression demonstrated biased learning of social evaluations about the self, suggesting that maladaptive social evaluation learning may represent a transdiagnostic vulnerability for social anxiety and depression. Advancing our understanding of the relevant cognitive mechanisms, our study highlighted the role of trait-like beliefs in these conditions. Whereas social anxiety was predominantly characterized by better learning of negative evaluations, depression may also involve a slower updating of all self-beliefs due to blunting of social information. Personalizing treatment with respect to these mechanisms may be beneficial.

## Methods

The Psychology Research Ethics Committee at the University of Bath approved the study, and the experiment was performed in line with institutional review board guidelines. Participants gave informed consent before the study. The Preregistered dataset was pre-registered on Open Science Framework (https://osf.io/ke3d5).

### Participants

The Mega-analysis collated data from 450 participants across nine studies (SI Study characteristics, Table A1). All studies recruited participants from the local community, although inclusion criteria and recruitment methods varied. Four studies recruited participants based on levels of social anxiety or depression symptoms. The remaining studies recruited healthy volunteers. If a study included multiple testing sessions, we included only data from the first session to reduce practice effects. Additionally, for two studies that used pharmacological manipulations we used only data from control conditions.

For the Preregistered dataset, we recruited 1062 participants aged 18 to 65 who were current residents of the United Kingdom using the online recruitment platform Prolific. We restricted participants to native English speakers without any literacy difficulties to ensure task comprehension. We recruited only individuals who had completed 5 or more Prolific studies with a ≥ 98% acceptance rate to ensure data quality. Of these, 836 (79%) participants completed the full study. We had to exclude 5 participants (0.6%) because of failed attention checks and 24 (3%) participants because of technical errors, leaving a final sample of 807.

Participants in the Preregistered dataset were on average older, and a lower proportion were female and students. Depression and social anxiety severity were similar between datasets (Table 1).

### Social evaluation learning

We measured learning of social evaluations using a two-alternative forced choice associative computer-based task. Participants were asked to learn whether the computer liked the self or a fictional other based on the computers’ feedback (task characteristics are outlined in SI Study characteristics, Table A1). In each trial, participants selected between a positive or negative social evaluative word and afterwards received feedback about the ‘correct’ evaluation (Fig. 1). The probability of ‘correct’ feedback following a positive evaluation varied between the three rules: In the positive, ‘Like’ rule, the positive social evaluative word was ‘correct’ in 60–80% of the trials depending upon the study; in the negative, ‘Dislike’ rule, the positive social evaluative word was ‘correct’ in only 20–40% of the trials. In the ‘neutral’ rule, selecting the positive social evaluative word was followed by ‘correct’ in 50% of the trials. At the end of each referential condition-rule block participants rated how much the computer liked the person being learnt about (0–10). All studies in the Mega-analysis were assessed under the supervision of researchers in psychology labs. The order of the rules was counterbalanced in each referential condition. For the Preregistered dataset participants completed the study procedure remotely using online survey ^20^ and experimental software ^21^. In the Preregistered dataset, participants completed either all self-referential conditions or all other-referential conditions first. The order of the positive, neutral, and negative rule in each referential condition was randomly determined.

### Self-report measure of mood and personality

We measured depression severity using the PHQ-9, a nine-item measure of DSM-IV depression symptoms within the previous two weeks^40^. The PHQ-9 has good psychometric properties and is widely used in clinical settings and the general population^54,55^. We measured social anxiety using the Brief Fear of Negative Evaluation Scale (BFNE)^56^. The BFNE is a 12-item measure of fear or worries in social contexts and has been validated for use in community and clinical samples^41,57^. Average PHQ scores, as reported in Table 1, were higher compared to representative studies in US samples^58^ and a higher amount of participants met cut-off criteria for major depressive disorder (Mega-analysis: 20.9%, Pre-registered: 25.3% at a cut-off score ≥ 10 where 10 is typically reported as the 95th percentile). Average BFNE scores were close to previously reported scores (*M* = 35.7, *SD* = 8.1^18^), .

### Statistical analyses

We measured participants’ ability to learn the positive and negative rules by calculating the average number of errors made before eight-consecutive rule-congruent responses (errors to criterion) for each referential-condition rule. Greater errors to criterion indicate worse learning. Optimistic bias for each referential condition was then calculated as the errors to criterion in the negative rule minus errors to criterion in the positive rule. Greater optimistic bias scores indicate relatively better learning of the positive relative to the negative rule. As the number of trials varied across studies in the Mega-analysis from 20 to 32, all analyses covering only the Mega-analysis included random effects for participants clustered within studies. We included participants as a random effect for all analysis in the Preregistered dataset.

We firstly evaluated if learning of social evaluations differed by referential condition and rule, irrespective of depression and anxiety. For this purpose, we conducted a mixed-effects linear regression model on errors to criterion predicted by referential condition, rule, and an interaction term between referential condition and rule.

We tested the preregistered hypothesis *H1.1* that *depression and social anxiety will be associated with better learning of negative relative to positive social evaluations about the self* using structural equation modelling. Optimistic bias scores for the self and other condition were entered as correlated predictor variables with PHQ-9 and BFNE scores as the correlated outcome variables. This model allowed us to examine the individual relationships between social evaluation learning with both depression and social anxiety whilst accounting for symptomatic comorbidity.

A second structural equation model tested the nested hypothesis H1.2 that *social anxiety will be associated with better learning of a negative ‘dislike’ rule about the self*,* whereas depression will be associated with impaired learning of a positive ‘like’ rule about the self.* Errors to criterion in each of the referential condition-rule combinations were entered as correlated predictor variables with PHQ-9 and BFNE scores as the correlated outcome variables. This model allowed us to examine the mechanisms underlying the relationship between social evaluation learning, depression, and social anxiety.

### Computational models

We described social evaluative learning with two distinct classes of reinforcement learning models: Associative learning and belief updating (see SI: Computational modelling of social evaluative learning for full details). Associative learning proposes that feedback enables individuals to associate values, here the perceived value of themselves and others, with states *s* and actions *a*. Belief updating models represent beliefs about the world, here about social evaluations, as probability distributions. Receiving positive (vs. negative) social evaluations serves as a reward that individuals seek out or avoid. Both model classes allow to distinguish initial trait beliefs, that is a general tendency to select the positive evaluation, from biased updating, that is differences in learning rates (in associative models) or updating and decay rates (in belief-based models).

Associative and belief-based learning models predict for each trial *t* how likely an individual chooses one action *a*_*t*_ with the probability *π*(*a*_*t*_; *s*_*t*_). Here, the actions consist of selecting a positive (a+) or negative (a-) evaluation. The probability to choose action *a*_*t*_, *π*(*a*_*t*_; *s*_*t*_), depends on their action values *Q*_*t*_*(a*_*t*_, *s*_*t*_*)* in state *s*_*t*_ and the trait belief, that is, the general tendency $\:{b}_{a+,s}.$ to select the positive evaluation,

1 $$\:{\uppi\:}\left({a}_{t}\:;{s}_{t}\right)=\:\frac{1}{1+{e}^{{Q}_{t}\left({a+}_{t},{s}_{t}\right)-{Q}_{t}\left(-,{s}_{t}\right)+{b}_{a+,s})}}$$

The distinct states *s*_*t*_ refer to each distinct experimental condition that the participant encounters. In the best associative and belief-based model, this tendency varied between the self- and the other condition, that is, we estimated the trait belief $\:{b}_{a+,s}$ as $\:{b}_{Self}$ in the self-referential condition and as $\:{b}_{Other}$ in the other-referential conditions.

In the most successful associative learning model (see SI Figure A7, Confirmation Positivity Bias SO Model), feedback $\:{r}_{t}$ after each action $\:{a}_{t}$ is used to update the action values for the chosen action for the next trial *Q*_*t+1*_*(a*_*t+1*_, *s*_*t+1*_*).*

2 $$\:{Q}_{t+1}\left({a}_{t+1},{s}_{t+1}\right)={Q}_{t}\left({a}_{t},{s}_{t}\right)+{\lambda\:}_{a,s}({r}_{t}-{Q}_{t}\left({a}_{t},{s}_{t}\right))$$

The model allowed individuals to learn at different rates depending on whether the evaluation matched (or contradicted) their own prediction. If participants received confirmatory feedback in trial *t*, the learning rate $\:{\lambda\:}_{a,s}$ was estimated as $\:{\lambda\:}_{Conf+}$; if participants received disconfirmatory feedback in trial *t*, the learning rate $\:{\lambda\:}_{a,s}$ was estimated as $\:{\lambda\:}_{Conf-}$.

Belief updating models, in contrast, propose that individuals form beliefs about themselves and their environment. The strength of beliefs *Q*_*t*_*(a*_*t*_, *s*_*t*_*)* increases as more evidence accumulates that supports them. In the most successful belief-based model (see SI Figure A7, Decay Trait Beliefs SO Model), feedback $\:{r}_{a,t}$ after each action updates the strength of the belief *Q*_*t*_*(a*_*t*_, *s*_*t*_*)* for the next trial *t* + 1:

3 $$\:{Q}_{t+1}\left({a}_{t+1},{s}_{t+1}\right)={\left(1-\eta\:\right)\:Q}_{t}\left({a}_{t},{s}_{t}\right)\:+\eta\:+\lambda\:\:{r}_{a,t}$$

The learning rate $\:\lambda\:$ modulates the strength of evidence from a new evaluation, $\:{r}_{a,t},$ and describes how strongly individuals updated their beliefs after receiving new evidence. The decay rate $\:\eta\:$ instead modulates the strength of evidence from previously held beliefs,$\:{\:Q}_{t}\left({a}_{t},{s}_{t}\right)$, and describes how fast evidence from previously held beliefs decay in memory (or vice versa, still exerts influence on the updated beliefs).

In an initial search for plausible mechanisms underpinning social evaluation learning, we identified the best associative learning model, the best belief updating model, and the globally best reinforcement learning model among all plausible candidates. Computational parameters were validated against errors to criterion and optimistic bias scores in a principal component factor analysis. We tested the hypothesis *H2.1* that *computational parameters*,* especially learning rates*,* reflect learning differences in depression and social anxiety* using a structural equation modelling framework. Model selection indicated that belief updating models fared overall better than associative learning models. Further, it was unnecessary to estimate separate learning and decay rates for the self and other condition in the best belief updating model. We therefore explored whether *depression (or social anxiety) biased the accumulation of social evidence about self and others”* (H2.2). Learning parameters, $\:\lambda\:$ and $\:\eta\:$, and the trait beliefs for the self and other condition ($\:{b}_{Self}$ and $\:{b}_{Other}$) were entered as correlated predictor variables with PHQ-9 and BFNE scores as the correlated outcome variables. Correlations between model parameters were low to moderate with the highest correlation between trait beliefs for self and others (Megaanalysis: *r* = .33; all other correlations r’s ≤ 0.15; Preregistered: *r* = .46; all other correlations ≤ 0.18). This model allowed us to examine the individual relationships between the mechanisms underpinning social evaluation learning with depression and social anxiety whilst accounting for symptomatic comorbidity.

## Electronic supplementary material

Below is the link to the electronic supplementary material.

Supplementary Material 1

## Data Availability

All data needed to evaluate the conclusions in the paper are present in the paper and/or the Supplementary Materials. This study was pre-registered on Open Science Framework (https://osf.io/ke3d5), where study materials, data for the Preregistered dataset and code are also made publicly available (https://osf.io/utyw5/). Data for the Mega-analysis dataset is available upon request as participants did not provide informed consent to publish data as open access.
