# Supplementary material for: Lack of optimistic bias during social evaluation learning reflects reduced positive self-beliefs in depression and social anxiety, but via distinct mechanisms
Source: Sci Rep. 2024 Sep 28;14:22471. doi: 10.1038/s41598-024-72749-6 (PMC11438955; doi:10.1038/s41598-024-72749-6)
Supplement: Supplementary file 1 — Supplementary Material 1 [file 41598_2024_72749_MOESM1_ESM.pdf]

Supplemental Information: Study characteristics

Table A1

Characteristics of study populations and the social evaluation learning task in the Mega-analysis and the Preregistered Study

Dataset	Recruitment	N	Inclusion Criteria	Demographics	Social Evaluation Learning Task				
					Referential Conditions	Feedback Contingencies	Trials Block	Total Trials	Test Sessions*
**Mega-analysis**									
Button et al., (2015) [1]	Social Anxiety: Low (BFNE £ 31), Moderate, or High (BFNE ³ 46)	100	Aged 18-50, no current psychiatric medication, English as a first language.	Gender, Age, Ethnicity, Employment, PHQ-9, BFNE	Self, Other	Positive (80%), Neutral (50%), Negative (20%)	32	192	1
Button, (unpublished a) [2]	Social Anxiety: Low (BFNE £ 26) or High (BFNE ³ 50)	48	Aged 18-50, English as a first language.	Gender, Age, Employment, PHQ-9, BFNE	Self, Other	Positive (80%), Negative (20%)	32	128	1
Button et al., (2016) [3] [a]	Community	48	Aged 18-50, English as a first language, no significant current or previous medical or psychiatric illness.	BFNE	Self, Other	Positive (80%), Negative (20%)	32	128	1[a]
Button, (unpublished b) [4]	Community	53	Aged 18-50, English as a first language.	Gender, Age, Ethnicity, PHQ-9, BFNE	Self, Other	Positive (80%), Negative (20%)	32	192	1
Brennan-Craddock & Button (unpublished) [5]	Community	17	Aged ³ 16, English as a first language.	Gender, Age, Employment, PHQ-9, BFNE	Self, Other	Positive (60%, 70%, 80%), Negative (20%, 30%, 40%)	20	240	1

Brennan-Craddock et al., (unpublished a) [6][c]	Community	8	Aged 18-35, normal or corrected-to-normal vision, right-handed.	Gender, Age, Employment, PHQ-9, BFNE	Self, Other	Positive (80%), Neutral (50%), Negative (20%)	32	192	3
Brennan-Cradock et al., (unpublished b) [6] [c]	Community	11	Aged 18-35, normal or corrected-to-normal vision, right-handed.	Gender, Age, Employment, PHQ-9, BFNE	Self, Other	Positive (80%), Neutral (50%), Negative (20%)	32	192	3
Hobbs et al., (2021) [7][c]	Depression: None (PHQ £ 5), Mild, or High (PHQ ³ 10)	144	Aged 18-65, normal or corrected-to-normal vision, fluent in English.	Gender, Age, Ethnicity, Employment, PHQ-9, BFNE	Self, Friend[c], Stranger	Positive (60%, 80%), Negative (20%, 40%)	24	288	2
Hobbs et al., (2020) [8][a]	Community	21	Aged 18-45, normal or corrected-to-normal vision, fluent in English, no significant current or previous medical or psychiatric illness, no current or past drug or alcohol dependency, no current use of psychoactive medication, no current pregnancy or breastfeeding, ≤ 5 cigarettes per day, ≤ 6 caffeinated drinks per day, lactose intolerance, no recreational psychoactive drug use within previous 3 months.	Gender, Age, Ethnicity, Employment, PHQ-9, BFNE	Self, Friend[c], Stranger	Positive (60%, 80%), Negative (20%, 40%)	24	288	1

**Preregistered**									
	Online	807	Aged 18-65, current resident in the UK, English as a first-language, no literacy difficulties, ^{3} 5 Prolific studies completed with ^{3} 98% acceptance rate.	Gender, Age, Ethnicity, Employment, PHQ-9, BFNE, Income, GAD, RSES, NPI, IPIP, PTS, BSQ, SCS, PSS, VAS	Self, Other	Positive (80%), Neutral (50%), Negative (20%)	32	192	1

[a] We only included data from the control (placebo) conditions because these studies used pharmacological manipulations.

[b] We only included data from the first testing session if the study had multiple testing sessions.

[c] Data from the 'friend' condition was not included in dataset 1.

PHQ-9 = 9-item Patient Health Questionnaire; BFNE = Brief Fear of Negative Evaluation Scale; GAD = Generalised Anxiety Disorder Assessment, RSES = Rosenberg Self-Esteem Scale, NPI = Narcissistic Personality Inventory, IPIP = Mini-International Personality Item Pool, PTS = Paranoid Thoughts Scale, BSQ = Body Shape Questionnaire, SCS = Self-Consciousness Scale, PSS = Perceived Stress Scale, VAS = Visual Analogue Scale for Mood

Table A2

Results from a Mixed ANOVA on Errors to Criterion with Condition (Self-referential vs. Other-referential) and Rule (Positive vs. Negative) as Within-Factors and Study as Between-Factor (Dataset Mega-analysis) by Dataset.

**Mega-analysis (n = 450)**	*df*	*MSE*	*F*	Partial η^2	*p*
Study	8, 441	39.0	6.3	0.103	<.001
Rule	1, 441	30.9	28.4	0.060	<.001
Study x Rule	8, 441	30.9	1.5	0.026	0.161
Condition	1, 441	14.9	< 1.0	<.001	0.905
Study x Condition	8, 441	14.9	1.4	0.024	0.207
Rule x Condition	1, 441	19.4	< 1.0	0.002	0.331
Study x Rule x Condition	8, 441	19.4	< 1.0	0.017	0.481
**Preregistered (n = 807)**	*df*	*MSE*	*F*	Partial η^2	*p*
Rule	1, 806	65.2	198.5	0.198	<.001
Condition	1, 806	28.0	7.1	0.009	.008
Rule x Condition	1, 806	28.9	13.5	0.016	<.001

Figure A1.

Errors to Criterion in each Study of the Mega-analysis (separate plots) depending upon Condition (Referent Self vs. Referent Other) and Rule (Positive vs. Negative).

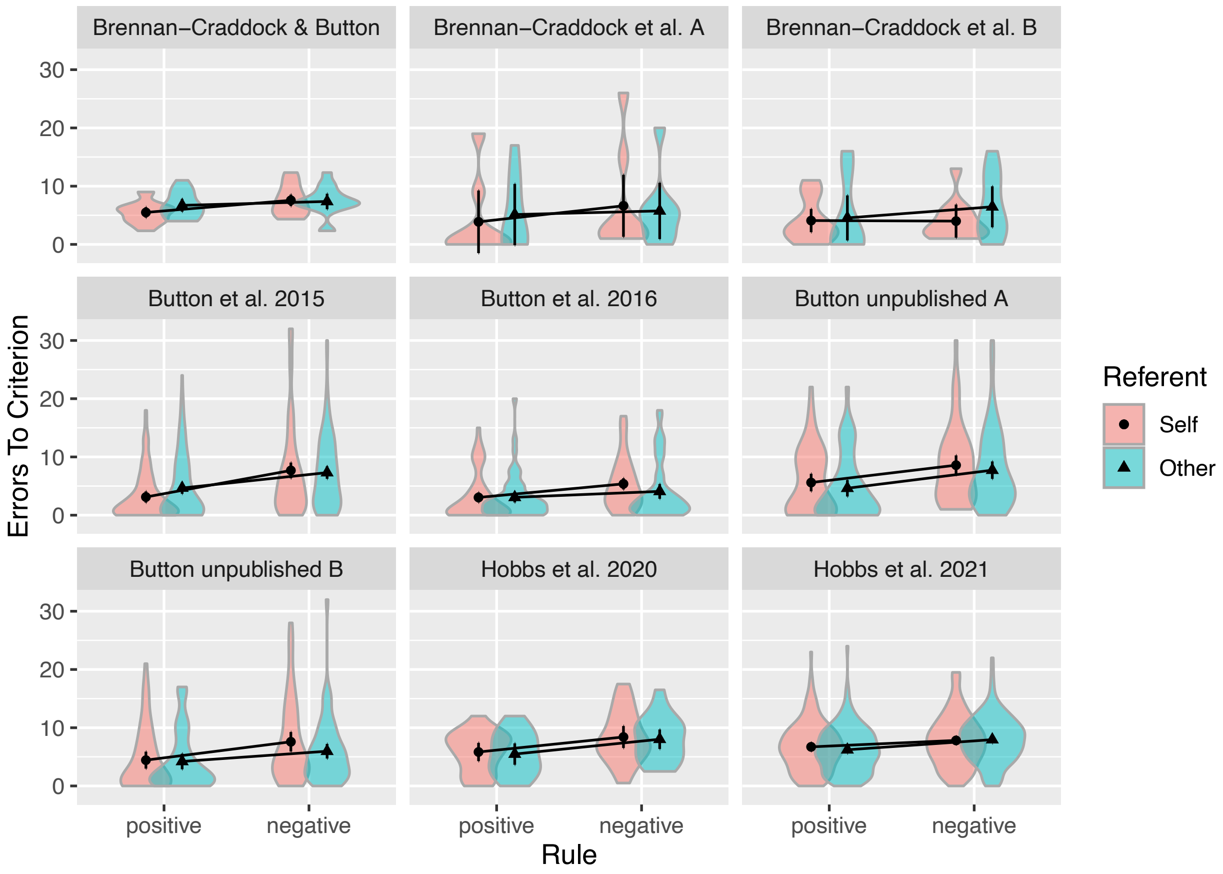

Figure A2.

Errors to Criterion in the Preregistered Study depending upon Condition (Referent Self vs. Referent Other) and Rule (Positive vs. Negative).

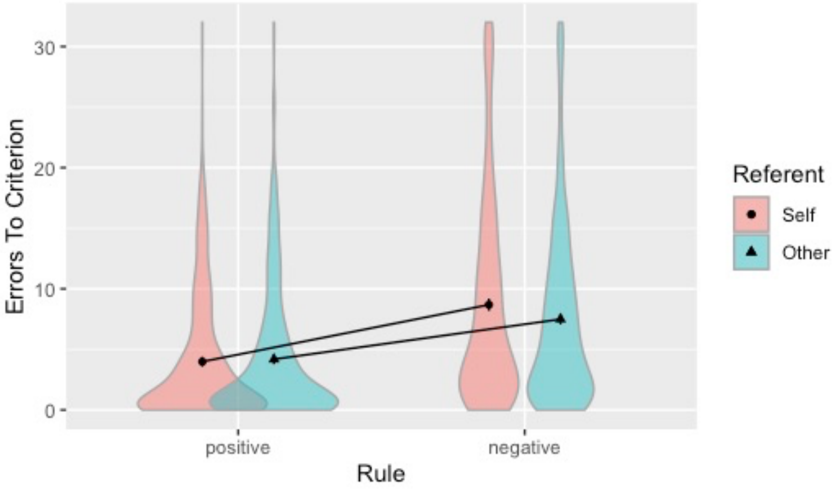

Supplemental Information: Social evaluation and psychopathological traits

Table A3

Structural equation models reporting regression pathways between social evaluation learning task measures (Optimistic Bias, Errors to Criterion) and symptoms (depression, PHQ-9, and social anxiety, BFNE) and correlations between depression (PHQ-9) and social anxiety (BFNE) by dataset

	Mega-analysis (n = 450)			Preregistered (n = 807)		
**Bias Scores**						
	ß	*95% CI*	*p*	*ß*	*95% CI*	*p*
PHQ-9						
Self	-0.25	-0.34, -0.15	< .001	-0.10	-0.18, - 0.03	.007
Other	0.01	-0.09, 0.10	.881	-0.02	-0.09, 0.06	.601
BFNE						
Self	-0.19	-0.28, -0.10	< .001	-0.13	-0.20, -0.05	< .001
Other	0.04	-0.05, 0.13	0.409	0.07	-0.00, 0.15	.062
Correlation	*r*	95% CI	*p*	*r*	95% CI	*p*
PHQ-9 ↔ BFNE	.39	.31, .47	< .001	.41	.35, .47	< .001
**Errors to Criterion**						
	ß	*95% CI*	*p*	*ß*	*95% CI*	*p*
PHQ-9						
Self-Positive	0.28	0.19, 0.38	< .001	0.06	-0.01, 0.13	.093
Self-Negative	-0.10	-0.20, -0.00	.048	-0.07	-0.15, 0.00	.056
Other-Positive	-0.02	-0.11, 0.08	.768	-0.01	-0.08, 0.06	.766
Other-Negative	-0.00	-0.11, 0.10	.938	-0.04	-0.11, 0.04	.363
BFNE						
Self-Positive	0.09	-0.01, 0.20	.067	0.03	-0.04, 0.10	.414
Self-Negative	-0.17	-0.27, -0.07	.001	-0.12	-0.20, -0.05	.001
Other-Positive	-0.03	-0.13, 0.07	.611	-0.08	-0.15, -0.004	.038
Other-Negative	0.03	-0.07, 0.13	.518	0.03	-0.04, 0.11	.384
Correlation	*r*	95% CI	*p*	*r*	95% CI	*p*
PHQ-9 ↔ BFNE	.40	.32, .48	< .001	.41	.35, .47	< .001

PHQ-9 = Patient Health Questionnaire; BFNE = Brief Fear of Negative Evaluation Scale. β = standardised path coefficient

Figure A3.

Relationship between optimistic bias and social anxiety (BFNE) in each study of the Mega-analysis (separate plots). A higher optimistic bias indicates that participants committed less errors when learning the positive relative to the negative rule.

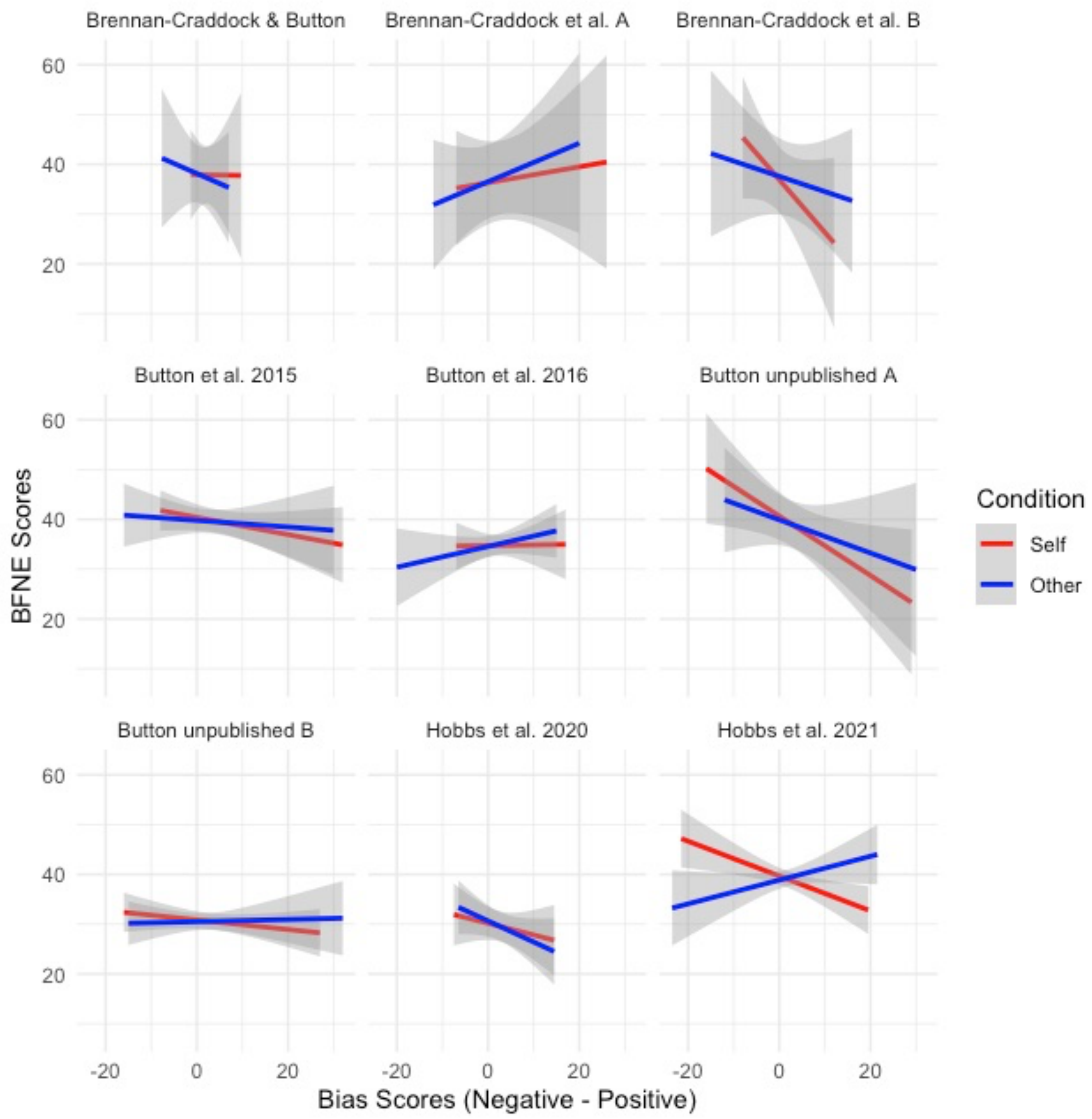

Figure A4.

Relationship between optimistic bias on the social evaluation learning task and depression (PHQ-9) in each study of the Mega-analysis (separate plots). A higher optimistic bias indicates that participants committed less errors when learning the positive relative to the negative rule.

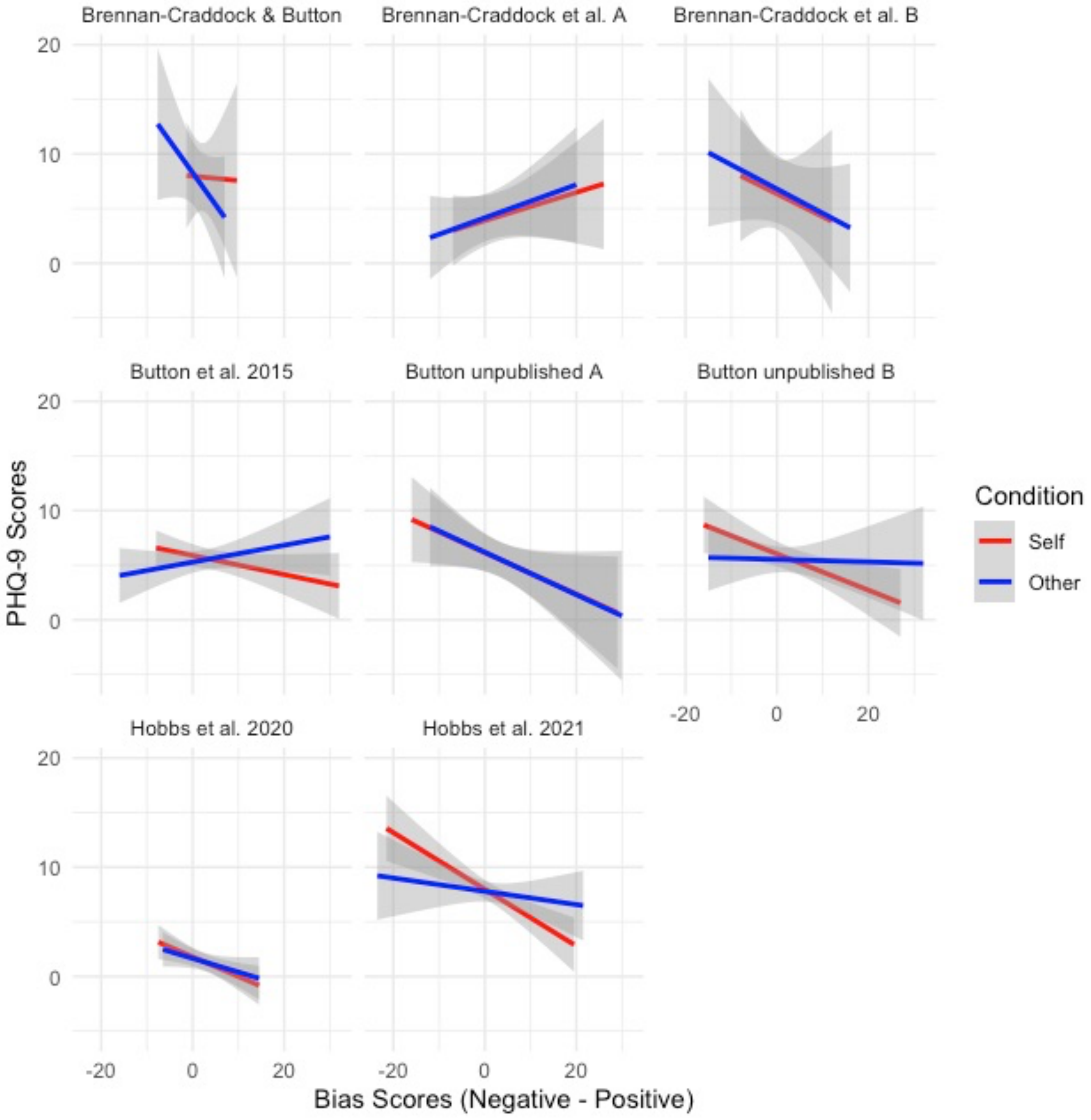

Supplemental Information: Computational modelling of social evaluative learning

To describe individual differences in social evaluative learning, we first considered two distinct classes of reinforcement learning models, associative and belief updating models. In a large initial model search step, we considered component mechanisms within each class that might best account for individual differences in social evaluative learning in the Mega-analysis. First, we split this data first into a training set (n = 361) and a validation set (n = 89). Second, we tested on the training data whether a model including one mechanism, such as memory decay, outperforms the 'baseline' model in its class, that is the model without that mechanism. We assessed further if the model parameters correlated substantially with psychopathological traits and if those correlations are consistent across models containing that parameter. Third, we evaluated if the training results replicated in the validation set and assessed in the validation data (a) if we could identify the same models as the best fitting ones and (b) if we could detect the same relationships of model parameters to psychopathological traits. Results are reported in this *SI* (section Results: Initial model search).

In the model evaluation step, we systematically combined these mechanisms within each class into six competitor learning models. We estimated the model parameters of all competitor learning models on the full Mega-analysis and determined the best associative learning model, the best belief updating model, and the best reinforcement learning model globally. Results are reported in this *SI* (section Results: Model evaluation).

In the model validation step, we validated the psychological plausibility of the model parameters against the descriptive learning indicators and tested their relationship to psychopathological traits. We assessed the construct validity of the model parameters with a principal component factor analysis on the model parameters and descriptive learning indicators, that is errors to criterion and optimistic bias measures. Next, we estimated the best learning models on the Preregistered dataset. Finally, we tested to what degree the previously identified relationships with depression and social anxiety replicated in the Preregistered dataset. We report results for the best belief updating model in the main text because belief updating models described social evaluations generally better than associative learning models (Results: Model evaluation, Figure A7). The identified relationships of model parameters with depression and social anxiety are, however, consistent across belief updating and associative learning models. Additional details are described in *SI Belief updating of social evaluations and psychopathological traits.* We report results for the best associative learning model in *SI Associative learning of social evaluations and psychopathological traits.*

Reinforcement learning models

Reinforcement learning models [9, 10] propose that individuals choose in each trial *t* probabilistically between *k* actions *a*, here a positive or a negative evaluation, depending upon the respective action values $Q_t(a_t, s_t)$ in state s_t.

$$\pi(a_t\,; s_t) = \frac{1}{1+e^{Q_t(a+_t, s_t) - Q_t(-, s_t) + b_{a+,s})}} \qquad (1)$$

$\pi(a_t; s_t)$ provides the probability of choosing action a_t, here selecting a positive (a+) or negative (a-), evaluation, in state s_t from their action values $Q_t(a_t, s_t)$ with $b_{a+,s}$ representing a general tendency to select the positive evaluation, that is a positivity bias (or a positive trait belief in belief updating). The states *s* here refer to the combination of the social evaluation rule used by the computer (positive, negative, or neutral) and whether the computer evaluated the participant or someone else (self or other referential condition).

Associative learning models. Associative learning models [9, 10] propose that the feedback $r_{a,t}$ updates the action values for the chosen action for the next trial $Q_{t+1}(a_{t+1}, s_{t+1})$.

$$Q_{t+1}(a_{t+1}, s_{t+1}) = Q_t(a_t, s_t) + \lambda_{a,s}(r_t - Q_t(a_t, s_t)) \qquad (2)$$

The learning rates $\lambda_{a,s}$ determine the degree to which individuals update the action values of each action in light of the feedback $r_{a,t}$. The feedback can take values of 1 ('choice was correct'), 0 (no feedback), or -1 ('choice was wrong'). $\lambda_{a,s} = 0$ indicates no updating; $\lambda_{a,s} = 1$ indicates a purely feedback-based switching between actions. Initial biases for one action compared to the other can be included in the model by setting $Q_1(a_1, s_1)$ equal to a free parameter $Q_0(a_0, s_0)$. Note that here the subscript *s* can refer to specific states; learning rates, response tendencies, and initial biases can differ between these states. For example, there may be a different learning rate following 'choice was right' than 'choice was wrong'.

All estimated associative learning models were nested within this general model, but used different learning rates, different initial biases, or positivity biases to describe social evaluative learning. Specifically, we allowed the learning rates to vary as a function of the action chosen or the current state. The simplest associative learning model (Simple RL) estimated only one learning rate for all states and actions. The valence model estimated a separate learning rate for a positive or a negative word (that is, a positive or negative evaluation by the computer, irrespective of whether the preceding choice was correct). The confirmation bias model estimated a separate learning rate depending upon whether the evaluation matched or mismatch the chosen action. The self-other models (S-O) proposed a different

learning rate for the self- and the other-referential condition, resulting in two learning rates when combined with the Simple RL model and in four learning rates when combined with the valence model. A final model combined the valence model with the confirmation bias resulting again in four separate learning rates. The initial bias model estimates an initial bias for the positive action in both conditions (Initial Bias) or a separate initial bias in the self and the other condition (Initial Bias S-O). Finally, the positivity bias encapsulates the idea that others often hold favourable opinions and thus choose the positive action more often in both conditions (Positivity Bias) or the positivity bias may vary depending upon whether oneself or another person is evaluated (Positivity Bias S-O).

Belief updating models. Within the very broad belief-based class, we specifically considered evidence accumulation models [9]. Belief updating models propose that agents hold beliefs about the world and update these beliefs with new incoming information. Here, the belief updating models assume that individuals accumulate evidence for each option in every trial t, but imperfectly, subject to an updating parameter λ, analogous to the learning rate above. Hence, we propose that individuals base their choice for action a in each trial t probabilistically on the amount of evidence accumulated for each option, $Q_t(a_t, s_t)$. After each action, feedback $r_{a,t}$ adds to the evidence $Q_t(a_t, s_t)$ for the next trial $t + 1$ according to:

$$Q_{t+1}(a_{t+1}, s_{t+1}) = (1 - \eta)\, Q_t(a_t, s_t) + \eta + \lambda\, r_{a,t} \tag{3}$$

with $r_{a,t}$ taking the value of 1 if the feedback provides new evidence for action a and 0 if it doesn't. The updating rate λ determines the strength of evidence accrued whenever r=1, whereas η describes the decay of the evidence in memory. Note that in the absence of any evidence, Q decays to the value of 1, not zero. Initial beliefs can be expressed by setting $Q_1(a_1, s_1)$ to a free parameter $Q_0(a_0, s_0)$. Initial beliefs are equivalent to trait beliefs without a memory decay.

All estimated belief updating models were nested within this more general one, but allowed the updating rate, the decay rate, or the trait belief to vary between the self- and the other-referential condition. Specifically, the Simple Belief Updating Model (Simple BUP) estimated one value of λ for both types of evaluations, whereas the Belief Updating Self-Other Model (BUP S-O) estimated a different updating rate for the self- and the other-referential condition. The two Asymmetric Updating (AU) models varied the updating rates λ between the positive compared to the negative action. The BUP AU assumed the same λ in the self- and the other-referential condition, whereas BUP AU S-O assumed separate λ in the self- and the other-referential condition (BUP AU S-O). The Trait Belief model specified a general preference for the positive over negative evaluation (Trait Belief) which could vary for the self-

and the other-referential condition (Trait Belief S-O). The Decay Model allowed memory strength to decay, postulating the same decay of beliefs across all states (Decay), a separate decay for the self or another person (Decay S-O), or a separate decay of the positive and the negative option (Decay AU).

Model estimation and comparison

The parameter values of all parameters within each model were estimated using maximum likelihood estimation, that is in model estimation we minimized the deviance -2LL, the negative summed log-likelihood L of the model given the data,

$$-2LL = -2\sum \ln(L)$$

We calculated the likelihood from the model-predicted probability $\pi(a_t; s_t)$ of choosing action a_t in state s_t and trial t for the participants' chosen action a_t.

We evaluate the models' relative performance within each model class, associative learning and belief updating models, with two different model fit indicators that balance model fit and generalizability. Akaike's Information Criterion (AIC) and the Bayesian Information Criterion (BIC [11]). AIC and BIC penalize more complex models by accounting for the number of free parameters with smaller AIC and BIC values indicating a better model fit. AICs and BICs were converted into differences, Δ AIC_M and Δ BIC_M , between the model M and the baseline learning models (Simple RL and Simple BUP, respectively). Additionally, we calculated AIC (BIC) model weights [12], $AIC_{w,M}$ or $BIC_{w,M}$, as relative fit indicators because datasets varied in the number of trials, conditions, and blocks.

Results: Initial model search

Associative learning models. Among the associative learning models, the confirmation model and the positivity bias model best described participants' learning of social evaluations in the Mega-analysis (Figure A5). The valence and the initial bias model fared well on the training set but did not outperform the Simple RL model in terms of BIC in the validation set. S-O models containing different learning rates for the self- and other referential condition (Simple RL S-O and valence S-O) also fared worse than the Simple RL model with respect to the BIC. Allowing for a positivity bias generally improved model performance compared to the Simple RL model as measured with AIC and BIC in the training and the validation set. Across all models containing a positivity bias, the positivity bias was consistently negatively associated with depression (Table A5) and, to a smaller extent, with social anxiety (Table A4). Correlational patterns were – as would be expected - similar for the initial bias. It was less clear if it is necessary to allow the positivity (or initial) bias to vary between the self- and the other-referential condition because AIC and BIC did not yield clear-cut results. However, as BIC only decreased slightly

and correlations remained stable, we preferred the more complex models that varied positivity (or initial) bias between the self- and other-referential condition. Finally, although models with a confirmation bias fared well in training and validation for both AIC and BIC, learning rates in the confirmation model did not reliably correlate with either social anxiety or depression. In contrast, the learning rates in the valence model correlated with psychopathological traits, in particular the differences between learning rates for positive and negative feedback in the self-referential condition (valence S-O).

Belief updating models. Among the belief updating models, the decay model including trait beliefs fared best overall in describing social evaluations in the Mega-analysis (Figure A6). On average, all trait belief models, that is models with a general preference for the positive evaluation outperformed the Simple BUP in training and validation on the AIC and BIC. Trait beliefs also correlated consistently with depression (Table A7), but not with social anxiety (Table A6). It was less clear if it was necessary to estimate separate trait beliefs in the self- and the other-referential condition. AIC suggested a considerably improved model fit of the Trait beliefs SO model, whereas BIC was slightly lower compared to the Trait beliefs model. However, as BIC only decreased slightly and correlations remained stable, we concluded that belief updating models with separate trait beliefs in the self- and the other-referential condition would provide a more complete account of social evaluation learning. Model fit was not consistently improved by separate updating rates in the self- and other-referential condition. Models with an asymmetric updating of beliefs or memory decay improved model fit beyond the simple BUP model with one updating rate. Interestingly, however, neither updating rates nor decay parameters were consistently associated with depression or social anxiety. Only the difference in asymmetric updating correlated slightly with depressive symptoms.

Results: Model evaluation

In the model evaluation step, we further determined the best learning model within each class, associative learning and belief updating, as well as the overall best reinforcement learning model. To this goal, we systematically combined the most promising learning mechanisms into six competitor learning models within each class. All reinforcement learning models encompassed a tendency to choose the positive over the negative evaluation, that is a positivity bias or trait beliefs. In associative learning, we compared a confirmation model with (or without) an initial bias, a valence model with (or without) an initial bias, and a valence model with different learning rates for the self- and other referential condition and with (or without) an initial bias. Positivity and initial biases were allowed to vary between the self- and the other-referential condition. In belief updating, we compared a decay model with (or without) initial beliefs, an asymmetric decay model with (or without) initial beliefs, and a decay model with (or without) initial beliefs. Trait and initial beliefs were allowed to vary between the self- and the other-

referential condition. All competitor models were first evaluated on the Mega-analysis dataset and we then assessed if the results replicated in the Preregistered dataset.

Figure A7 displays model fits for all associative learning and belief updating models as AIC and BIC differences to the Simple RL model, separately for the Mega-analysis (n = 450, A, C) and the Preregistered dataset (n = 807, B, D). Belief updating models consistently outperform associative learning models across both datasets and model fit indicators and hence better describe how individuals learn to infer social evaluations from feedback. The best competitor in both classes, however, depends on the fit measure used.

Among the associative learning models, model fit indicators suggested that the confirmation models (with and without initial bias) on average better described participants' social evaluation learning than the valence models across both data sets. It was less clear whether an initial bias towards the positive option was necessary to successfully describe and predict social evaluation: Models proposing an initial bias had a higher average AIC_w across participants (Mega-analysis: M =.57, SD = .31; Preregistered: M =.57, SD = .31) than models without an initial bias (Mega-analysis: M = .43, SD = .31; Preregistered: M =.43, SD = .31), but the BIC_w was lower (Mega-analysis: M =.22, SD = .34; Preregistered: M =.22, SD = .33) than for models without an initial bias (Mega-analysis: M =.78, SD = .34; Preregistered: M =.78, SD = .33). To conclude, the best supported associative learning model, the confirmation-positivity model, encompasses separate positivity biases for the self- than the other-referential condition and separate learning rates after confirmatory and disconfirmatory feedback.

Among the belief updating models, model fit indicators suggested that belief updating models with a decay of memory traces described social evaluation learning consistently better across both data sets than models without memory decay or models proposing an asymmetric updating of beliefs. Considering changeable initial beliefs in the model did not improve model fit: Models proposing an initial bias on average had a lower AIC_w (Mega-analysis: M =.36, SD = .23; Preregistered: M =.38, SD = .24) than models without an initial bias (Mega-analysis: M = .64, SD = .23; Preregistered: M =.62, SD = .24). BIC_w were also lower (Mega-analysis: M =.06, SD = .12; Preregistered: M =.06, SD = .14) than for models without an initial bias (Mega-analysis: M =.94, SD = .12; Preregistered: M =.94, SD = .14). To conclude, the best supported belief updating model, the decay-trait belief SO model, encompasses separate trait beliefs for the self- than the other-referential condition and a separate mechanism for updating beliefs and memory decay.

In the remainder, we focus our main analysis on this decay-trait belief SO model and report additional details in *SI Belief updating of social evaluations and psychopathological traits.* We report matching results for the best associative learning model, the confirmation-positivity model, in *SI Associative learning of social evaluations and psychopathological traits.*

Figure A5

Model fits for the initial associative learning models in the training set (n = 361, A, C) and the validation set (n = 89, B, D) of the Mega-analysis dataset. Model fits are measured as AIC (A, B) and BIC differences (C, D) to the Simple RL model. Lower AIC and BIC differences indicate a better model fit. Bar charts indicate that associative learning models with a positivity bias, a separate positivity bias for the self and the other person, or a confirmation bias emerge as the best models.

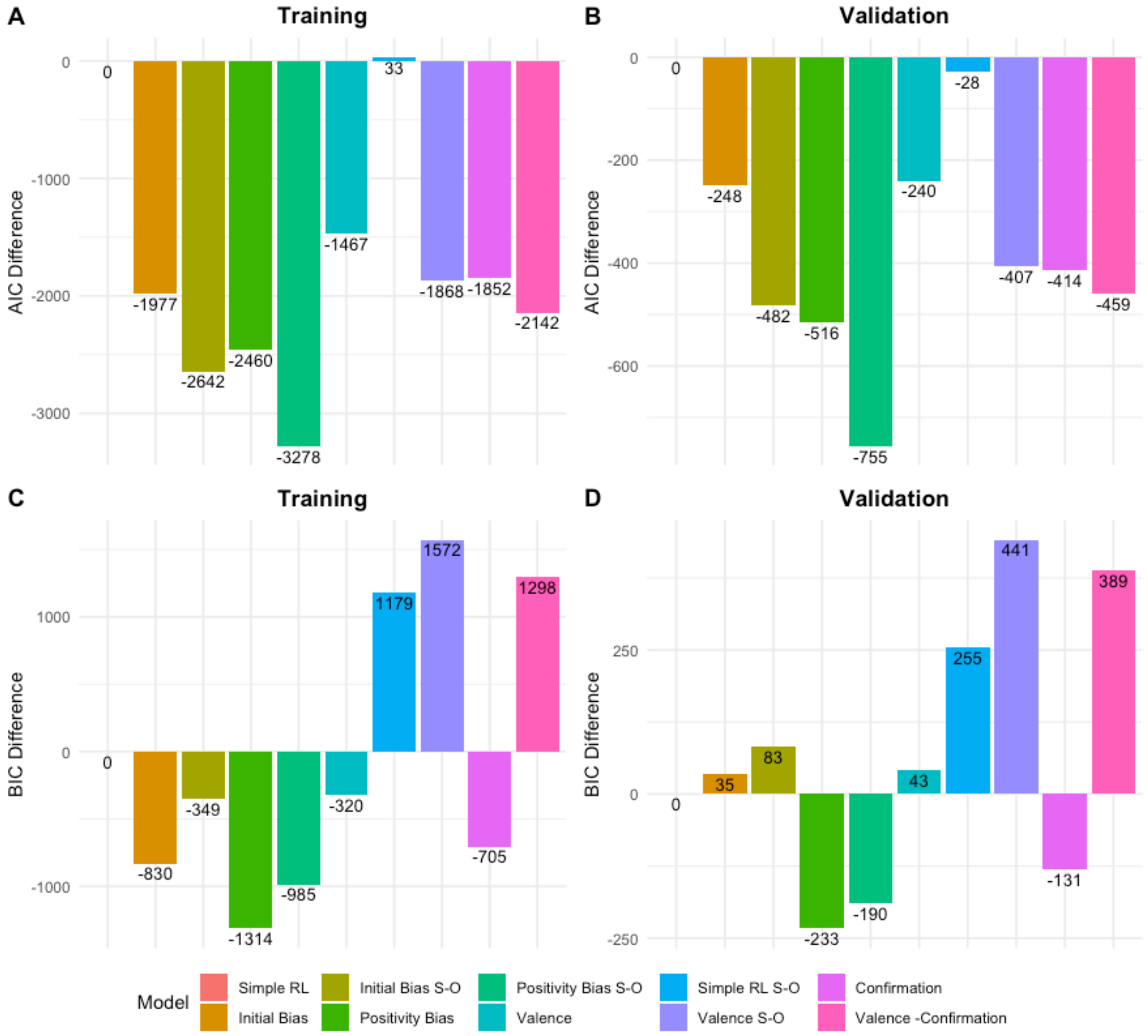

Figure A6

Model fits for the initial belief updating models in the training set (n = 361, A, C) and the validation set (n = 89, B, D) of the Mega-analysis dataset. Model fits are measured as AIC (A, B) and BIC differences (C, D) to the Simple BUP model. Lower AIC and BIC differences indicate a better model fit. Bar charts indicate belief updating models with separate trait beliefs for the self and the other person, a memory decay, or asymmetric belief updating emerge as the best models.

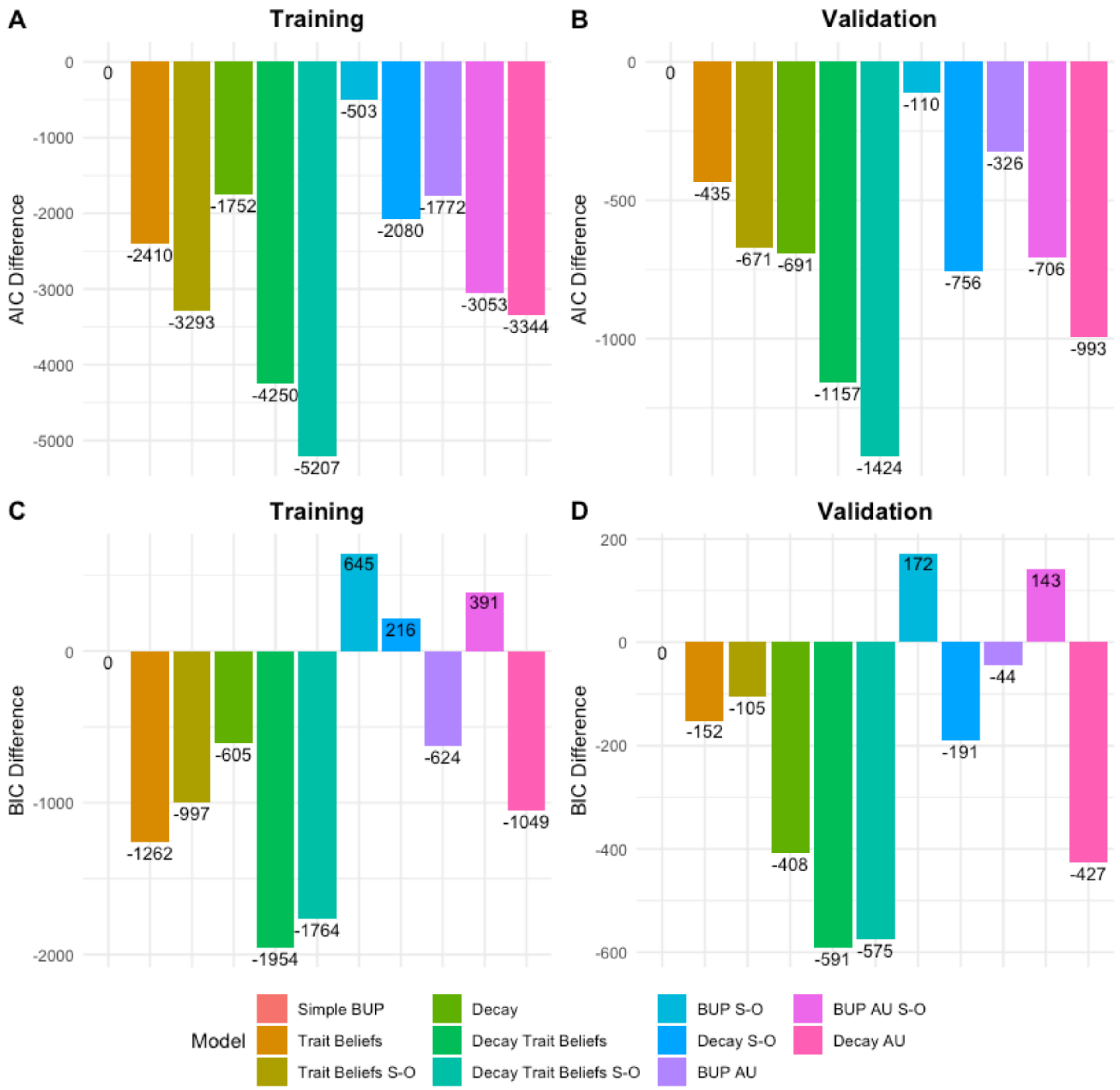

Table A4

Correlations between model parameters and social anxiety (BFNE) for each associative learning model in the Mega-analysis (upper row: Training, lower row: validation)

r BFNE	λ_{a+}	λ_{a-}	$\Delta\lambda_{a}$	λ_{Self}	λ_{Other}	$\Delta\lambda_{Self}$	λ_{Conf}	λ_{DConf}	$\Delta\lambda_{Conf}$	$Q_{0,Self}$<br>b_{Self}	$Q_{0,Other}$<br>b_{Other}	ΔQ_0<br>Δb
Simple RL	-0.01											
	-0.03											
Initial Bias	0.01									-0.15		
	-0.04									-0.10		
Initial Bias	0.03									-0.18	-0.03	-0.14
S-O	-0.05									-0.08	0.05	-0.11
Positivity Bias	0.00									-0.16		
	-0.03									-0.10		
Positivity Bias	-0.02									-0.16	-0.04	-0.13
S-O	-0.02									-0.12	0.07	-0.15
Valence	-0.09	0.07	-0.16									
	-0.12	0.03	-0.16									
Simple RL S-O				-0.06	0.01	-0.05						
				-0.11	0.09	-0.16						
	$\lambda_{a+,Self}$	$\lambda_{a-,Self}$	$\Delta\lambda_{a,Self}$	$\lambda_{a+,Other}$	$\lambda_{a-,Other}$	$\Delta\lambda_{a,Other}$						
Valence S-O	-0.15	0.09	-0.21	-0.06	-0.04	-0.02						
	-0.18	0.05	-0.19	0.11	0.02	0.1						
Confirmation							-0.06	0.01	-0.06			
							-0.12	0.12	-0.18			
	$\lambda_{a+,Conf+}$	$\lambda_{a-,Conf}$	$\Delta\lambda_{a,Conf}$				$\lambda_{a+,DConf}$	$\lambda_{a-,DConf}$	$\Delta\lambda_{a,DConf}$			
Valence -	-0.12	-0.01	-0.09				-0.04	0.01	-0.06			
Confirmation	-0.12	-0.10	-0.01				0.02	0.09	-0.06			

Note: λ_{a+} = Learning rate for positive words; λ_{a-} = Learning rate for negative words; $\Delta\lambda_a = \Delta\lambda_{a+} - \Delta\lambda_{a-}$
λ_{Self} = Learning rate in the self-referential condition; λ_{Other} = Learning rate in the other-referential condition; $\Delta\lambda_{self} = \Delta\lambda_{Self} - \Delta\lambda_{Other}$
λ_{Conf} = Learning rate after confirmatory feedback; λ_{DConf} = Learning rate after disconfirmatory feedback, $\Delta\lambda_{Conf} = \Delta\lambda_{Conf} - \Delta\lambda_{DConf}$
Q_0 = Initial bias in the respective condition (self or other-referential); $\Delta Q_0 = Q_{0,Self} - Q_{0,Other}$
b = Positivity bias in the respective condition (self or other-referential); $\Delta b = b_{Self} - b_{Other}$

Table A5

Correlations between model parameters and depression (PHQ) for each associative learning model in the Mega-analysis (upper row: Training, lower row: validation)

r PHQ	λ_{a+}	λ_{a-}	$\Delta\lambda_{a}$	λ_{Self}	λ_{Other}	$\Delta\lambda_{Self}$	λ_{Conf}	λ_{DConf}	$\Delta\lambda_{Conf}$	$Q_{0,Self}$ b_{Self}	$Q_{0,Other}$ b_{Other}	ΔQ_{0} Δb
Simple RL	-0.14											
	-0.03											
Initial Bias	-0.13									-0.19		
	-0.06									-0.24		
Initial Bias	-0.14									-0.22	-0.06	-0.15
S-O	-0.02									-0.31	-0.1	-0.25
Positivity Bias	-0.17									-0.24		
	-0.03									-0.32		
Positivity Bias	-0.19									-0.26	-0.09	-0.19
S-O	-0.07									-0.25	-0.21	-0.12
Valence	-0.26	-0.04	-0.24									
	-0.17	0.14	-0.34									
Simple RL S-O				-0.11	-0.15	0.02						
				-0.05	0.04	-0.07						
	$\lambda_{a+,Self}$	$\lambda_{a-,Self}$	$\Delta\lambda_{a,Self}$	$\lambda_{a+,Other}$	$\lambda_{a-,Other}$	$\Delta\lambda_{a,Other}$						
Valence S-O	-0.24	0.08	-0.27	-0.2	-0.12	-0.11						
	-0.18	0.12	-0.25	-0.1	0.15	-0.26						
Confirmation							-0.15	-0.12	-0.03			
							-0.03	0.09	-0.09			
	$\lambda_{a+,Conf}$	$\lambda_{a-,Conf}$	$\Delta\lambda_{a,Conf}$				$\lambda_{a+,DConf}$	$\lambda_{a-,DConf}$	$\Delta\lambda_{a,DConf}$			
Valence -	-0.22	-0.02	-0.16				-0.13	-0.06	-0.08			
Confirmation	-0.12	0.09	-0.15				0.05	0.13	-0.07			

Note: λ_{a+} = Learning rate for positive words; λ_{a-} = Learning rate for negative words; $\Delta\lambda_{a} = \Delta\lambda_{a+} - \Delta\lambda_{a-}$
λ_{Self} = Learning rate in the self-referential condition; λ_{Other} = Learning rate in the other-referential condition; $\Delta\lambda_{self} = \Delta\lambda_{Self} - \Delta\lambda_{Other}$
λ_{Conf} = Learning rate after confirmatory feedback; λ_{DConf} = Learning rate after disconfirmatory feedback, $\Delta\lambda_{Conf} = \Delta\lambda_{Conf} - \Delta\lambda_{DConf}$
Q_0 = Initial bias in the respective condition (self or other-referential); $\Delta Q_0 = Q_{0,Self} - Q_{0,Other}$
b = Positivity bias in the respective condition (self or other-referential); $\Delta b = b_{Self} - b_{Other}$

Table A6

Correlations between model parameters and social anxiety (BFNE) for each belief updating model in the Mega-analysis (upper row: Training, lower row: validation)

r BFNE	λ_{Self}	λ_{Other}	$\Delta\lambda_{Self}$	η_{Self}	η_{Other}	$\Delta\eta_{Self}$	$Q_{0,Self}$ b_{Self}	$Q_{0,Other}$ b_{Other}	ΔQ_0 Δb
Simple BUP	0.00								
	-0.05								
Trait Beliefs	-0.01						-0.14		
	-0.08						-0.09		
Trait Beliefs S-O	-0.01						-0.16	-0.01	-0.15
	-0.08						-0.10	0.04	-0.12
Decay	-0.09			-0.10					
	0.07			0.08					
Decay Trait Beliefs	-0.11			-0.11			-0.13		
	0.06			0.08			-0.08		
Decay Trait Beliefs S-O	-0.11			-0.11			-0.16	0.00	-0.16
	0.09			0.08			-0.09	0.05	-0.12
BUP S-O	0.04	-0.05	0.08						
	-0.16	0.01	-0.16						
Decay S-O	-0.09			-0.10	-0.05	-0.04			
	0.04			0.05	0.07	0.00			
	$\lambda_{AU+,Self}$	$\lambda_{AU-,Self}$	$\Delta\lambda_{AU,Self}$	$\lambda_{AU+,Other}$	$\lambda_{AU-,Other}$	$\Delta\lambda_{AU,Other}$			
BUP AU	-0.05	0.00	-0.07						
	-0.10	-0.09	-0.05						
BUP AU S-O	-0.04	0.04	-0.10	-0.08	-0.1	0.01			
	-0.16	-0.13	-0.07	0.13	0.05	0.10			
	λ			η_{AU+}	η_{AU-}	$\Delta\eta_{AU}$			
Decay AU	-0.11			-0.04	-0.14	0.10			
	0.05			0.09	0.1	-0.06			

Note: λ = Updating rate (if only λ_{Self} is estimated, $\lambda_{Self} = \lambda_{Other}$) ; η = Decay rate (if only η_{Self} is estimated, $\eta_{Self} = \eta_{Other}$)
λ_{Self} = Updating rate in the self-referential condition; λ_{Other} = Updating rate in the other-referential condition; $\Delta\lambda_{Self} = \Delta\lambda_{Self} - \Delta\lambda_{Other}$;
η_{Self} = Updating rate in the self-referential condition; η_{Other} = Updating rate in the other-referential condition; $\eta_{Self} = \Delta\eta_{Self} - \Delta\eta_{Other}$;
Q_0 = Initial bias in the respective condition (self or other-referential); $\Delta Q_0 = Q_{0,Self} - Q_{0,Other}$
λ_{AU} = Asymmetric updating rate, $\Delta\lambda_{AU,Self} = \lambda_{AU+,Self} - \lambda_{AU-,Self}$
b = Positivity bias in the respective condition (self or other-referential); $\Delta b = b_{Self} - b_{Other}$

Table A7

Correlations between model parameters and depression (PHQ) for each belief updating model in the Mega-analysis (upper row: Training, lower row: validation)

r PHQ	λ_{Self}	λ_{Other}	$\Delta\lambda_{\text{Self}}$	η_{Self}	η_{Other}	$\Delta\eta_{\text{Self}}$	$Q_{0,\text{Self}}$ b_{Self}	$Q_{0,\text{Other}}$ b_{Other}	ΔQ_0 Δb
Simple BUP	-0.15								
	0.04								
Trait Beliefs	-0.16						-0.24		
	0.01						-0.29		
Trait Beliefs S-O	-0.16						-0.25	-0.10	-0.17
	-0.01						-0.22	-0.23	-0.08
Decay	-0.15			-0.02					
	0.06			0.01					
Decay Trait Beliefs	-0.18			0.03			-0.24		
	0.02			0.01			-0.30		
Decay Trait Beliefs S-O	-0.17			-0.01			-0.25	-0.10	-0.17
	0.01			0.01			-0.22	-0.24	-0.07
BUP S-O	-0.16	-0.06	-0.1						
	0.00	-0.06	0.04						
Decay S-O	-0.16			0.02	-0.05	0.06			
	0.04			-0.09	0.12	-0.17			
	$\lambda_{\text{AU+,Self}}$	$\lambda_{\text{AU-,Self}}$	$\Delta\lambda_{\text{Self}}$	$\lambda_{\text{AU+,Other}}$	$\lambda_{\text{AU-,Other}}$	$\Delta\lambda_{\text{Other}}$			
BUP AU	-0.22	-0.07	-0.20						
	-0.10	0.03	-0.16						
BUP AU S-O	-0.24	-0.08	-0.20	-0.11	-0.06	-0.06			
	-0.09	0.07	-0.18	-0.15	0.01	-0.18			
	λ			$\eta_{\text{AU+}}$	$\eta_{\text{AU-}}$	$\Delta\eta_{\text{AU}}$			
Decay AU	-0.17			0.20	-0.06	0.18			
	0.01			0.07	-0.01	0.04			

Note: λ = Updating rate (if only λ_{Self} is estimated, $\lambda_{\text{Self}} = \lambda_{\text{Other}}$) ; η = Decay rate (if only η_{Self} is estimated, $\eta_{\text{Self}} = \eta_{\text{Other}}$)
λ_{Self} = Updating rate in the self-referential condition; λ_{Other} = Updating rate in the other-referential condition; $\Delta\lambda_{\text{Self}} = \Delta\lambda_{\text{Self}} - \Delta\lambda_{\text{Other}}$;
η_{Self} = Updating rate in the self-referential condition; η_{Other} = Updating rate in the other-referential condition; $\eta_{\text{Self}} = \Delta\eta_{\text{Self}} - \Delta\eta_{\text{Other}}$;
Q_0 = Initial bias in the respective condition (self or other-referential); $\Delta Q_0 = Q_{0,Self} - Q_{0,Other}$
λ_{AU} = Asymmetric updating rate, $\Delta\lambda_{\text{AU,Self}} = \lambda_{\text{AU+,Self}} - \lambda_{\text{AU-,Self}}$
b = Positivity bias in the respective condition (self or other-referential); $\Delta b = b_{Self} - b_{Other}$

Figure A7

Model fits for the final associative learning and belief updating models in the Mega-analysis (n = 450, A, C) and the Preregistered dataset (n = 807, B, D). Model fits are measured as AIC (A, B) and BIC differences (C, D) to the Simple RL model. Lower AIC and BIC differences indicate a better model fit. The clearest finding is that belief updating models describe social evaluation learning better than associative learning models, and this is robustly replicated across datasets and fit measures. Among the associative learning models, a model with a confirmation bias and a separate positivity bias for the self and other fares best. Among the belief updating models, a model with memory decay and separate trait beliefs about the self and the other person fares best. In both classes, the winning model depends on the fit measure used.

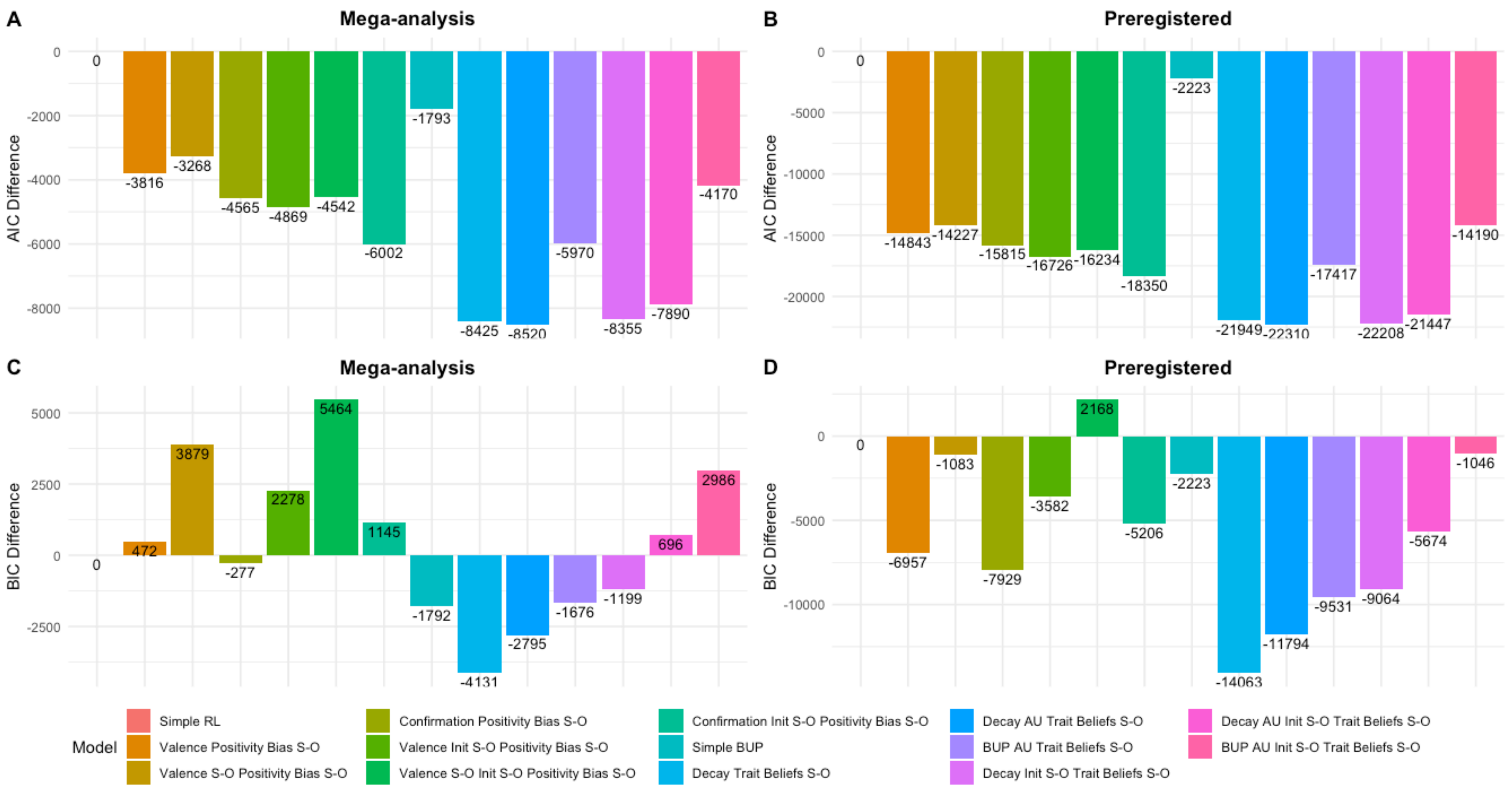
A
Mega-analysis
AIC Difference
0
-3816
-3268
-4565
-4869
-4542
-6002
-1793
-8425
-8520
-5970
-8355
-7890
-4170
B
Preregistered
AIC Difference
0
-14843
-14227
-15815
-16726
-16234
-18350
-2223
-21949
-22310
-17417
-22208
-21447
-14190
C
Mega-analysis
BIC Difference
0
472
3879
-277
2278
5464
1145
-1792
-4131
-2795
-1676
-1199
696
2986
D
Preregistered
BIC Difference
0
-6957
-1083
-7929
-3582
2168
-5206
-2223
-14063
-11794
-9531
-9064
-5674
-1046
Model
Simple RL
Valence Positivity Bias S-O
Valence S-O Positivity Bias S-O
Confirmation Positivity Bias S-O
Valence Init S-O Positivity Bias S-O
Valence S-O Init S-O Positivity Bias S-O
Confirmation Init S-O Positivity Bias S-O
Simple BUP
Decay Trait Beliefs S-O
Decay AU Trait Beliefs S-O
BUP AU Trait Beliefs S-O
Decay Init S-O Trait Beliefs S-O
Decay AU Init S-O Trait Beliefs S-O
BUP AU Init S-O Trait Beliefs S-O

Supplemental Information: Belief updating in social evaluation and psychopathological traits

Table A8

Component loadings from the PCA for the best belief updating model (Decay – Trait Beliefs SO) in the Mega-analysis

	Belief Updating			
	RC1	RC2	RC3	RC4
$\text{Err}_{\text{Self+}}$	-0.59		0.39	0.34
$\text{Err}_{\text{Self-}}$	0.78		0.33	0.27
$\text{Bias}_{\text{Self}}$	0.99			
b_{Self}	0.9			
$\text{Err}_{\text{Other+}}$		-0.57	0.58	
$\text{Err}_{\text{Other-}}$		0.73	0.52	
$\text{Bias}_{\text{Other}}$		0.99		
b_{Other}		0.88		
λ			-1.1	0.47
η			-0.45	1.11
Var %	0.28	0.26	0.19	0.14
Cum Var %	0.28	0.54	0.73	0.87

Notes: Err = errors to criterion in each referential-rule condition, Bias = optimistic bias scores; b_{Self} = Trait belief in the self-referential condition; b_{Other} = Trait belief in the other-referential condition; λ = Updating rate; η = Decay rate

Table A9

Correlations between social anxiety (BFNE) or depression (PHQ-9) with computational parameters estimates from the best belief updating model (Decay – Trait Beliefs SO) in the Mega-analysis and the Preregistered dataset. Social anxiety (BFNE) negatively correlated with beliefs about the self b_{Self}, modelled as trait beliefs in belief updating. PHQ-9 scores were associated with more negative beliefs about the self in the belief updating model, but additionally correlated with updating rate λ and more negative beliefs about other persons (b_{Other}) in the Mega-analysis.

	**Estimate**	**BFNE**		**PHQ-9**[a]	
**Parameter**	***M* (*SE*)**	***r***	***CI***	***r***	***CI***
		Mega-analysis (n = 450)			
λ	.51 (.01)	-.07	-.16, .02	-.14	-.23, -.04
η	.19 (.01)	-.07	-.16, .02	.00	-.10, .09
b_{Self}	0.29 (0.04)	-.15	-.24, -.06	-.25	-.34, -.15
b_{Other}	0.22 (0.03)	.01	-.09, .10	-.12	-.24, -.02
		Preregistered (n = 807)			
λ	.45 (.01)	.05	-.02, .12	-.03	-.10, .03
η	.19 (.01)	-.03	-.10, .04	-.02	-.09, .05
b_{Self}	0.48 (0.04)	-.08	-.15, -.01	-.12	-.19, -.05
b_{Other}	0.31 (0.03)	-.01	-.08, .06	-.07	-.14, .00

Notes: PHQ-9 = Patient Health Questionnaire; BFNE = Brief Fear of Negative Evaluation Scale. *r* = correlation coefficient; 95% CI = 95% confidence interval

b_{Self} = Trait belief in the self-referential condition; b_{Other} = Trait belief in the other-referential condition; λ = Updating rate; η = Decay rate

[a]PHQ-9 was not measured in all studies (n = 402).

Table A10

Results from structural equation models reporting regression pathways from computational parameters in the social evaluation learning task to psychopathological traits (depressive symptoms, PHQ-9, and social anxiety, BFNE) separately for the best belief updating model (Decay – Trait Beliefs SO) on the Mega-analysis and the Preregistered dataset.

**Parameters**	**Mega-analysis**			**Preregistered**		
Regression	*ß*	*95% CI*	*p*	*ß*	*95% CI*	*p*
PHQ-9						
λ	-0.15	-0.25, -0.05	.002	-0.04	-0.11, 0.03	.251
η	0.02	-0.07, 0.11	.640	0.01	-0.06, 0.08	.865
b_{Self}	-0.22	-0.31, -0.13	<.001	-0.11	-0.19, -0.03	.005
b_{Other}	-0.01	-0.11, 0.09	.787	-0.02	-0.10, 0.06	.560
BFNE						
λ	-0.06	-0.16, 0.03	.186	0.05	-0.02, 0.12	.125
η	-0.06	-0.15, 0.03	.198	-0.02	-0.09, 0.05	.516
b_{Self}	-0.18	-0.27, -0.08	<.001	-0.10	-0.18, -0.02	.013
b_{Other}	0.07	-0.03, 0.17	.166	0.05	-0.03, 0.12	.255
Correlation	*r*	95% CI	*P*	*r*	95% CI	*p*
PHQ-9 ↔						
BFNE	.40	.32, .48	<.001	.41	.35, .47	<.001

Notes: PHQ-9 = Patient Health Questionnaire; BFNE = Brief Fear of Negative Evaluation Scale. β = standardised path coefficient; 95% CI = 95% confidence interval

b_{Self} = Trait belief in the self-referential condition; b_{Other} = Trait belief in the other-referential condition; λ = Updating rate; η = Decay rate

Supplemental Information: Associative learning of social evaluation and psychopathological traits

Model evaluation identified the confirmation-positivity SO model as the best supported associative learning model, encompassing separate positivity biases for the self--referential b_{Self} than the other-referential condition b_{Other} and separate learning rates after confirmatory, λ_{Conf}, and disconfirmatory feedback, λ_{DConf}. This confirmation-positivity SO model reveals a stronger positivity bias, that is a preference for choosing the positive option, in the self than the other condition (Table A12). Learning rates indicate that individuals learn faster after receiving feedback confirming their initial choice than after receiving feedback that contradicts their choice. We compared this confirmation-positivity SO model against a valence based model. This valence positivity SO model assumed separate learning rates following positive and negative words in the self and other condition as well as distinct trait beliefs. Closer inspection of this model suggested that social anxiety correlated only negatively with positive trait beliefs about the self (r = -.09), whereas higher depressive symptoms were correlated negatively with positive trait beliefs about the self (r = -.11) and the other person (r = -.10). Learning rates were uncorrelated with social anxiety and depression. However, the confirmation-positivity SO model outperformed the valence positivity SO model in model selection (Figure A7), indicating that confirmatory vs. disconfirmatory feedback may play a larger role in social evaluation learning than valence-based updating of information. In the remainder, we focus our analysis therefore on this confirmation-positivity SO model.

We assessed the construct validity of the model parameters with a principal component factor analysis using errors to criterion and optimistic bias as additional independent variables (Figure A8, Table A11). The variability in model parameters and scores was sufficiently explained by a three-factor solution (73.8 % of the variance explained) according to eigenvalues and a parallel analysis. The positivity bias for the self b_{Self} primarily reflected the optimistic bias in the self-referential condition, whereas the positivity bias for others b_{Other} primarily reflected the optimistic bias in the other condition. The learning rate after confirmatory feedback λ_{Conf} reflected errors to criterion in all referential-condition rule combinations, that is, individuals with smaller learning rates made more errors until they reached the criterion for the positive and negative rule when learning about both the self and other. The learning rate after dis-confirmatory feedback λ_{DConf} did not find a corresponding match in task measures.

Finally, we determined to what degree computational parameters estimates from the best associative learning model are associated with psychopathological traits. Table A12 reports the zero-order correlations of social anxiety (BFNE) or depression (PHQ-9) with computational parameters estimates from the best associative learning model in the Mega-analysis and the Preregistered dataset. Replicating

the results from belief updating models, beliefs about the self, modelled as a positivity bias in associative learning, negatively correlated with social anxiety and depression. Depression was also associated with lower learning rates and more negative beliefs about other persons in the Mega-analysis, but not in the Preregistered study. Figure A9 displays those relationships and summarizes results from structural equation modelling of depressive symptoms and social anxiety, analogously to reported results for belief updating models in the main text. In line with hypothesis H2.1, the positivity bias for the self was robustly predictive of lower depressive symptoms and reduced social anxiety. We found partial support for the hypothesis H2.2. that individuals with higher depressive symptoms display a blunted response to social feedback as learning parameters were only predictive of depression in the Mega-analysis and not in the Preregistered dataset.

Figure A8. Left panel: Correlations between model parameters (λ_{Conf}, λ_{DConf}, b_{Self}, b_{Other}) of the associative learning model (Confirmation – Positivity SO), errors to criterion in each referential-rule condition (Err), and optimistic bias scores ($\text{Bias}_{\text{Self}}$, $\text{Bias}_{\text{Other}}$). Right panel: Factor loadings in a principal component analysis for the associative learning model (Confirmation – Positivity SO). Blue colours indicate positive correlations (loadings); red colours indicate negative correlations (loadings).

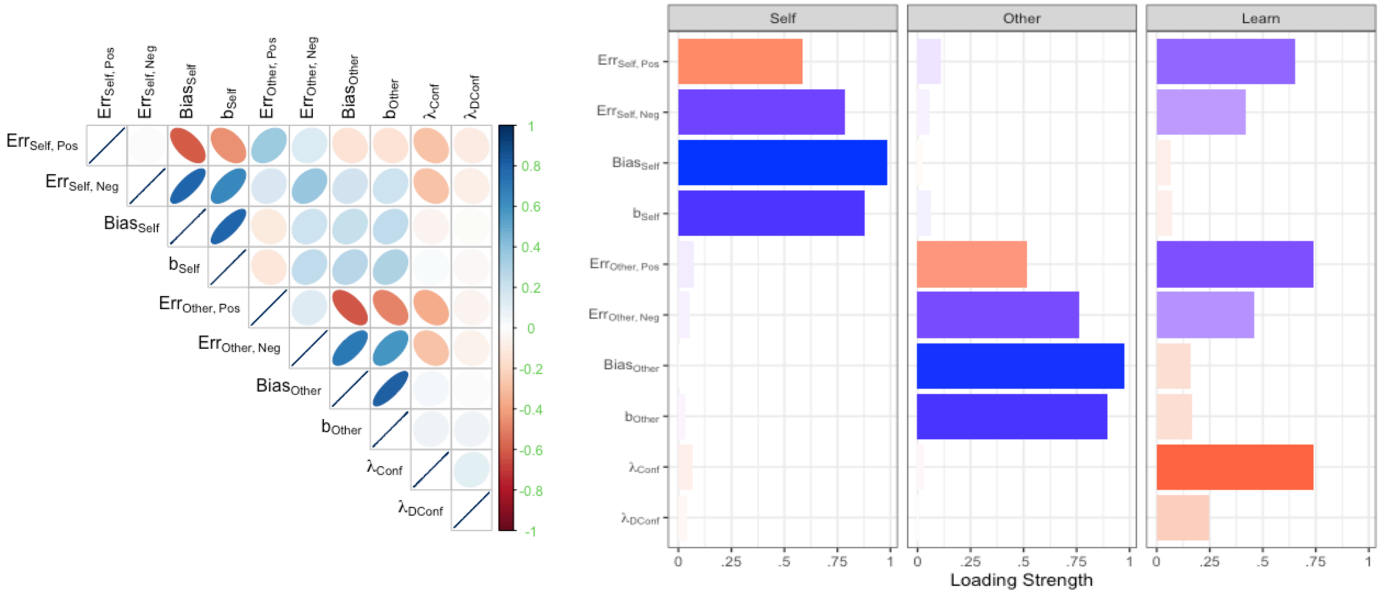

Table A11

Component loadings from the PCA for the best associative learning model (Confirmation – Positivity SO) in the Mega-analysis

	RC1	RC2	RC3
Err_{Self+}	-0.58		0.65
Err_{Self-}	0.79		0.42
$Bias_{Self}$	0.99		
b_{Self}	0.88		
Err_{Other+}		-0.51	0.74
Err_{Other-}		0.76	0.46
$Bias_{Other}$		0.97	
b_{Other}		0.9	
λ_{Conf}			-0.74
$\lambda_{DisConf}$			-0.25
Var %	0.27	0.26	0.2
Cum Var %	0.27	0.54	0.74

Notes: Err = errors to criterion in each referential-rule condition, Bias = optimistic bias scores; b_{Self} = Positivity bias in the self-referential condition; b_{Other} = Positivity bias in the other-referential condition; λ_{Conf} = Learning rate after confirmatory feedback; λ_{DConf} = Learning rate after disconfirmatory feedback

Table A12

Correlations between social anxiety (BFNE) or depression (PHQ-9) with computational parameters estimates from the best associative learning model (Confirmation - Positivity SO) in the Mega-analysis and the Preregistered dataset. Social anxiety (BFNE) negatively correlated with beliefs about the self b_{Self}*, modelled as a positivity bias in associative learning. PHQ-9 scores were associated with more negative beliefs about the self in associative learning in the Preregistered dataset, but additionally correlated with lower learning rates (*λ_{Conf}*,* λ_{DConf}*) and more negative beliefs about other persons (*b_{Other}*) in the Mega-analysis.*

	**Estimate**	**BFNE**		**PHQ-9**[a]	
**Parameter**	***M*** **(*SE*)**	***r***	***95% CI***	***r***	***95% CI***
		Mega-analysis (n = 450)			
λ_{Conf}	.57 (.01)	-.06	-.16, .03	-.14	-.24, -.05
λ_{DConf}	.37 (.01)	-.01	-.10, .08	-.12	-.22, -.02
b_{Self}	0.27 (0.04)	-.14	-.23, -.05	-.24	-.33, -.15
b_{Other}	0.20 (0.03)	-.01	-.11, .08	-.12	-.20, .00
		Preregistered (n = 807)			
λ_{Conf}	.53 (.01)	.01	-.06, .08	-.04	-.11, .03
λ_{DConf}	.31 (.01)	.02	-.05, .09	.02	-.05 .08
b_{Self}	0.42 (0.04)	-.08	-.15, -.02	-.10	-.17, -.03
b_{Other}	0.27 (0.03)	-.01	-.08, .06	-.07	-.14, .00

Notes: PHQ-9 = Patient Health Questionnaire; BFNE = Brief Fear of Negative Evaluation Scale. *r* = correlation coefficient; 95% CI = 95% confidence interval

b_{Self} = Positivity bias in the self-referential condition; b_{Other} = Positivity bias in the other-referential condition; λ_{Conf} = Learning rate after confirmatory feedback; λ_{DConf} = Learning rate after disconfirmatory feedback

[a]PHQ-9 was not measured in all studies (n = 402).

Figure A9

A. Relationship between associative learning parameters (Confirmation – Positivity SO) estimated in the social evaluation learning task and depressive symptoms (PHQ-9, i) and social anxiety (BFNE, ii). A higher positivity bias for the self b_{Self} (or the other, b_{Other}) indicate that participants believed others perceived them (or another person) positively. Higher learning rates after confirmatory feedback λ_{Conf} (or disconfirmatory feedback, λ_{DConf}) indicate that participants faster updated the action values for an option if their chosen option matched (mismatched) the feedback. A stronger positivity bias for the self b_{Self} was consistently associated with less depressive symptoms and less social anxiety. B. Structural equation modelling of depressive symptoms and social anxiety as predicted by computational parameters estimated within the associative learning model. Social anxious participants and depressed participants more likely held a positivity bias about the self b_{Self}, learning rates after confirmatory feedback λ_{Conf} and disconfirmatory feedback λ_{DConf} were not unambiguously associated with depressive symptoms.

A

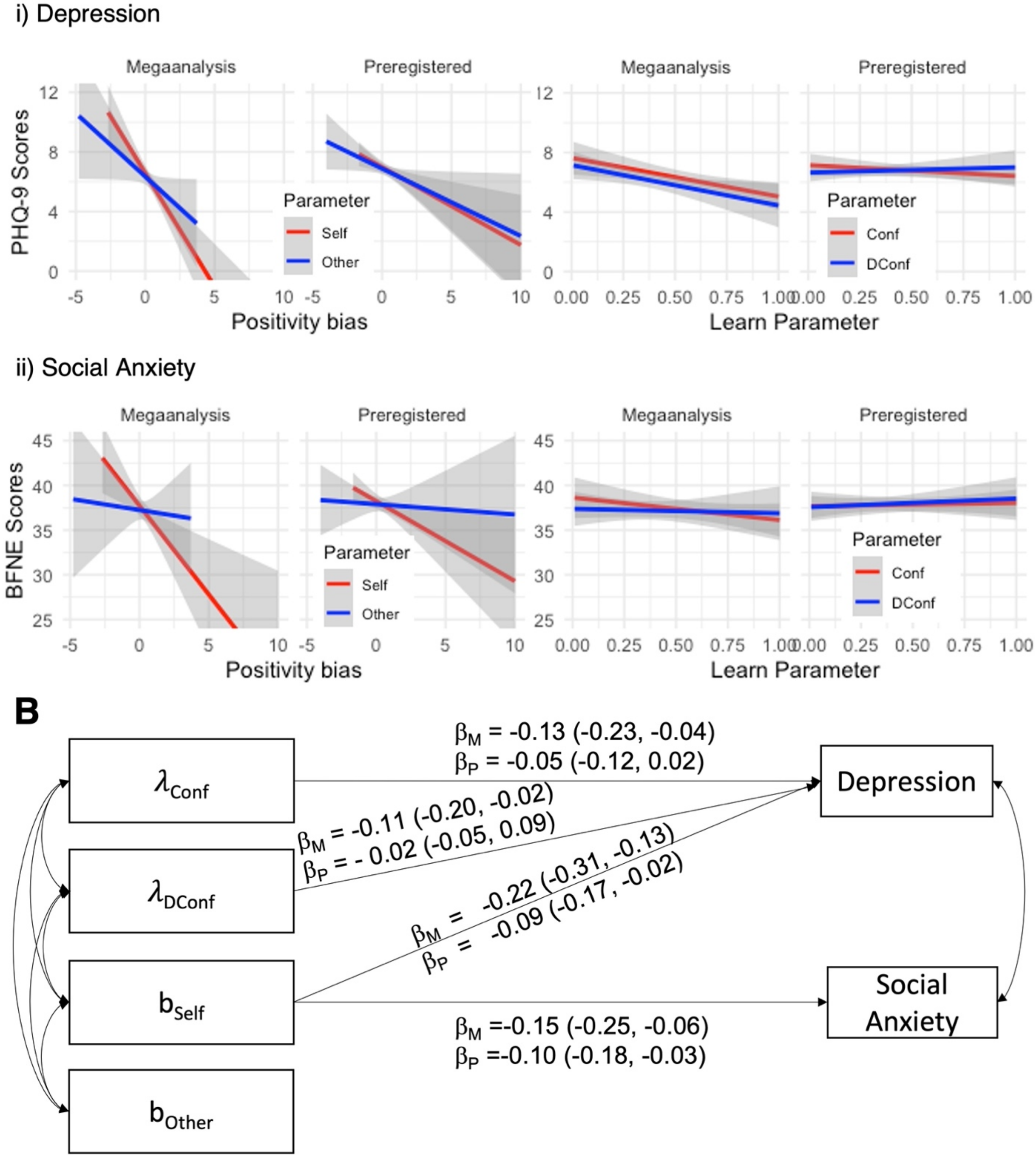

i) Depression
Megaanalysis
Preregistered
Megaanalysis
Preregistered
PHQ-9 Scores
Parameter
Self
Other
Parameter
Conf
DConf
Positivity bias
Learn Parameter
ii) Social Anxiety
Megaanalysis
Preregistered
Megaanalysis
Preregistered
BFNE Scores
Parameter
Self
Other
Parameter
Conf
DConf
Positivity bias
Learn Parameter
B
λ_{Conf}
λ_{DConf}
b_{Self}
b_{Other}
Depression
Social Anxiety
β_M = -0.13 (-0.23, -0.04)
β_P = -0.05 (-0.12, 0.02)
β_M = -0.11 (-0.20, -0.02)
β_P = - 0.02 (-0.05, 0.09)
β_M = -0.22 (-0.31, -0.13)
β_P = -0.09 (-0.17, -0.02)
β_M =-0.15 (-0.25, -0.06)
β_P =-0.10 (-0.18, -0.03)

Table A13

Results from structural equation models reporting regression pathways from computational parameters in the social evaluation learning task to psychopathological traits (depressive symptoms, PHQ-9, and social anxiety, BFNE) for the best associative learning (Confirmation – Positivity SO) on the Mega-analysis and the Preregistered dataset

**Parameters**		**Mega-analysis**			**Preregistered**	
Regression	*ß*	*95% CI*	*p*	*ß*	*95% CI*	*p*
PHQ-9						
λ_{Conf}	-0.13	-0.22, -0.04	.005	-0.05	-0.12, 0.02	.135
λ_{DConf}	-0.11	-0.20, -0.02	.021	0.02	-0.05, 0.09	.533
b_{Self}	-0.22	-0.31, -0.13	<.001	-0.09	-0.17, -0.02	.019
b_{Other}	0.00	-0.09, 0.10	.930	-0.03	-0.11, 0.05	.416
BFNE						
λ_{Conf}	-0.07	-0.16, 0.03	.158	0.00	-0.07, 0.07	.997
λ_{DConf}	-0.02	-0.11, 0.08	.713	0.02	-0.05, 0.09	.563
b_{Self}	-0.15	-0.25, -0.06	.002	-0.10	-0.18, -0.03	.009
b_{Other}	0.04	-0.04, 0.14	.386	0.04	-0.04, 0.12	.306
Correlation	*r*	95% CI	*P*	*r*	95% CI	*p*
PHQ-9 ↔						
BFNE	.40	.32, .48	<.001	.41	.35, .47	<.001

Notes: PHQ-9 = Patient Health Questionnaire; BFNE = Brief Fear of Negative Evaluation Scale. β = standardised path coefficient; 95% CI = 95% confidence interval

b_{Self} = Positivity bias in the self-referential condition; b_{Other} = Positivity bias in the other-referential condition; λ_{Conf} = Learning rate after confirmatory feedback; λ_{DConf} = Learning rate after disconfirmatory feedback

References

1. Button KS, Kounali D, Stapinski L, Rapee RM, Lewis G, Munafo MR. Fear of negative evaluation biases social evaluation inference: evidence from a probabilistic learning task. PLoS One. 2015;10(4):e0119456. doi: 10.1371/journal.pone.0119456.
2. Button KS. Identifying the Neural Substrates of Learning Social Evaluation in Social Anxiety. . Unpublished.
3. Button KS, Karwatowska L, Kounali D, Munafo MR, Attwood AS. Acute anxiety and social inference: An experimental manipulation with 7.5% carbon dioxide inhalation. J Psychopharmacol. 2016;30(10):1036-46. doi: 10.1177/0269881116653105.
4. Button KS. Self-referential learning and self-esteem. Unpublished.
5. Brennan-Craddock A, Button KS. Self-referential processing in Depression. Unpublished.
6. Brennan-Craddock A, Xin W, Sui J, Button KS. The Emotional Self. Unpublished.
7. Hobbs C, Sui J, Kessler D, Munafo MR, Button KS. Self-processing in relation to emotion and reward processing in depression. Psychol Med. 2021:1-13. doi: 10.1017/S0033291721003597.
8. Hobbs C, Murphy SE, Wright L, Carson J, Assche IV, O'Brien J, et al. Effect of acute citalopram on self-referential emotional processing and social cognition in healthy volunteers. BJPsych Open. 2020;6(6):e124. doi: 10.1192/bjo.2020.107.
9. Hopkins AK, Dolan R, Button KS, Moutoussis M. A Reduced Self-Positive Belief Underpins Greater Sensitivity to Negative Evaluation in Socially Anxious Individuals. Comput Psychiatr. 2021;5(1):21-37. doi: 10.5334/cpsy.57.
10. Sutton RS, Barto AG. Reinforcement learning: An introduction. MIT press; 2018.
11. Schwarz G. Estimating the dimension of a model. The Annals of Statistics. 1978;6:461-4.
12. Wagenmakers EJ, Farrell S. AIC model selection using Akaike weights. Psychon Bull Rev. 2004;11(1):192-6. doi: 10.3758/bf03206482.
